# Genomic architecture of 5S rDNA cluster and its variations within and between species

**DOI:** 10.1186/s12864-022-08476-x

**Published:** 2022-03-27

**Authors:** Qiutao Ding, Runsheng Li, Xiaoliang Ren, Lu-yan Chan, Vincy W. S. Ho, Dongying Xie, Pohao Ye, Zhongying Zhao

**Affiliations:** 1 Department of Biology, Hong Kong Baptist University, Hong Kong SAR, China; 2 Department of Infectious Diseases and Public Health, City University of Hong Kong, Hong Kong SAR, China; 3 State Key Laboratory of Environmental and Biological Analysis, Hong Kong Baptist University, Hong Kong SAR, China

**Keywords:** *Caenorhabditis elegans*, *C. briggsae*, 5S rDNA cluster, 5S, 18S-5.8S-26S, Oxford Nanopore technologies

## Abstract

**Background:**

Ribosomal DNAs (rDNAs) are arranged in purely tandem repeats, preventing them from being reliably assembled onto chromosomes during generation of genome assembly. The uncertainty of rDNA genomic structure presents a significant barrier for studying their function and evolution.

**Results:**

Here we generate ultra-long Oxford Nanopore Technologies (ONT) and short NGS reads to delineate the architecture and variation of the 5S rDNA cluster in the different strains of *C. elegans* and *C. briggsae*. We classify the individual rDNA’s repeating units into 25 types based on the unique sequence variations in each unit of *C. elegans* (N2). We next perform assembly of the cluster by taking advantage of the long reads that carry these units, which led to an assembly of 5S rDNA cluster consisting of up to 167 consecutive 5S rDNA units in the N2 strain. The ordering and copy number of various rDNA units are consistent with the separation time between strains. Surprisingly, we observed a drastically reduced level of variation in the unit composition in the 5S rDNA cluster in the *C. elegans* CB4856 and *C. briggsae* AF16 strains than in the *C. elegans* N2 strain, suggesting that N2, a widely used reference strain, is likely to be defective in maintaining the 5S rDNA cluster stability compared with other wild isolates of *C. elegans* or *C. briggsae*.

**Conclusions:**

The results demonstrate that Nanopore DNA sequencing reads are capable of generating assembly of highly repetitive sequences, and rDNA units are highly dynamic both within and between population(s) of the same species in terms of sequence and copy number. The detailed structure and variation of the 5S rDNA units within the rDNA cluster pave the way for functional and evolutionary studies.

**Supplementary Information:**

The online version contains supplementary material available at 10.1186/s12864-022-08476-x.

## Background

Ribosomal RNAs (rRNAs) as the components of ribosomes play a critical role in protein synthesis. Eukaryotic rRNAs are encoded by ribosomal DNAs (rDNAs) that are arranged in tandem repeats within the rDNA clusters. There are four rRNA genes, i.e., 5S rRNA, 18S rRNA, 5.8S rRNA, and 28S rRNA. The 5S rDNA cluster is usually arranged as tandem repeats that are separate from the remaining three genes in most species with a few exceptions, including yeast [1]. In *C. elegans*, each 5S rDNA repeating unit contains a 5S rRNA gene, a splicing leader gene (SL1), and two non-transcribed sequences (NTS). The 18S, 5.8S, and 28S rRNAs are produced as a single transcript using 45S rDNA as a template, which is also arranged as a tandem array in the genome. The transcript is processed into three individual RNAs following transcription [2, 3]. In contrast to most mRNAs and microRNAs that are produced with RNA polymerase II (Pol II) [4], the 45S rRNAs are transcribed by RNA polymerase I (Pol I), and the 5S rRNAs are transcribed by RNA polymerase III (Pol III) along with tRNA genes. Intriguingly, rRNAs made by RNA pol II from an artificial gene were able to rescue the phenotype of an rDNA deletion mutant in yeast [5], indicating that rRNAs transcribed by RNA pol II are functional. Yeast rDNA cluster contains both rDNA gene and a protein-coding gene, TAR1 [6], suggesting that RNA Pol I and II are functionally compatible. However, a functional study in yeast suggests that a specific chromatin structure in rDNA down-regulates polymerase II promoters [7]. In contrast, a transgene landed into the 28S rDNA in zebrafish is expressed [8]. Therefore, it remains unclear whether the genomic environment of rDNAs consisting of tandem repeats is permissive for overall mRNA transcription in all other species.

The rDNA copy number is known to be variable between cells, or individuals with different ages. For example, copy loss is a recurrent feature in cancers associated with mTOR activation [9]. rDNA copy number loss during aging has been in canine brain [10] and human blood cells [11]. The rDNA copy number variations (CNVs) between different wild isolates or mutated strains of *C. elegans* have been estimated by next-generation sequencing (NGS) reads and quantitative PCR [12, 13] to range from 33 to 418 copies for the 45S rDNA and from 39 to 438 copies for the 5S rDNA. However, the rDNA CNV during development has not been reported in *C. elegans* and other nematodes. In addition to CNV, sequence variation is also noted in the rDNAs from individuals of the same species [14, 15].

The sequences of rDNA genes and its non-transcribed sequences are found to have polymorphisms in eukaryotic species, including single-nucleotide polymorphisms (SNPs) and small insertions or deletions (INDELs). For example, in the mouse and human, the INDELs ranging from 1 to 12 bps in rDNA were frequently identified between chromosomes, tissues, individuals, and families [16, 17]. Similar polymorphisms in rDNA were also identified in yeast [18], fly [19] and plants [20]. Previous studies demonstrated that the *C. elegans* genome carried only a single type of 5S rDNA unit with few SNPs in its coding sequence [14, 21], whereas its related species, *C. briggsae*, carried two distinct types of 5S rDNA unit with the 5S rDNA gene arranged in the opposite orientation relative to splicing leader 1 (SL1) [22, 23]. Whether there are any 5S rDNA variants in the NTS region of nematode species has not been thoroughly investigated.

NGS techniques have been intensively used to assemble genomes across species in the past two decades, leading to an exponential increase of genomic data. However, the genome assembly produced with NGS reads only is usually poor in continuity due to the presence of repetitive sequences, especially in those regions consisting of highly tandem repeats such as centromeres and rDNAs. Therefore, these tandem repeats are commonly included in various contigs that are unable to be assigned to precise locations on chromosomes. The repetitive sequences create a huge challenge for genome assembly using NGS reads because of their relatively short read lengths usually ranging from 100 to 200 bps. Therefore, extra efforts have been made to improve the continuity of an assembly, including mate-pair sequencing of the ends from a large genomic fragment [24], incorporation of genetic markers [25] or chromatin configuration (Hi-C) [26], or using the long reads synthesized with the NGS short reads [27]. These steps have significantly improved the continuity of genome assemblies, especially for those relatively small genomes. *C. elegans’* isogenic genome is the first metazoan genome that was assembled using Sanger sequencing reads coupled with physical mapping, leading to an exceptionally high contiguity [25]. It barely contains any gaps except in the rDNA clusters and telomere sequences. However, the high mapping costs prevent the universal application of this approach to other species. The genome assembly of its companion species, *C. briggsae*, was generated using shotgun sequencing coupled with scaffolding with end sequencing of bacterial artificial chromosomes (BAC) and fosmids [23]. The resulting contigs or supercontigs were assembled onto chromosomes using genetic markers [28] or synthetic long reads (SLR) coupled with Hi-C [27]. However, these efforts failed to resolve the localization and genomic organization of rDNA clusters. Delineation of the genomic architecture and localization of rDNA clusters is needed for studying the evolution, function, and regulation of ribosomal genes [29–32].

Third-generation sequencing (TGS) techniques, including Oxford Nanopore Technologies (ONT) sequencing and PacBio Single Molecule, Real-Time (SMRT) sequencing, overcome the intrinsic limitation of the short-read by generating ultra-long reads with limited sequencing bias [33], which is expected to facilitate genome assembly with an improved continuity by the inclusion of more repetitive sequences [34–36]. Importantly, the amplification-free TGS enables researchers to directly sequence DNA or RNA with a reduced sequence bias [37]. Due to its ultra-long length, TGS reads have recently been used to re-sequence the *C. elegans* genome, which recovered substantially more repetitive sequences and revealed chromosomal rearrangements and structural variations between strains [34, 38, 39]. Recently, the TGS reads have been adopted to generate telomere-to telomere human genome with limited success [40, 41]. However, these reads have not been used to resolve the genomic structure of the 5S rDNA and 45S rDNA clusters in other species.

Here, we characterized the genomic architecture of the 5S rDNA cluster in both *C. elegans* and *C. briggsae* using both ONT sequencing and NGS reads. Aided by these reads, we identified various reproducible sequence variations in the 5S rDNA unit in both species, which allowed us to generate an assembly of 5S rDNA cluster carrying up to at least 167 consecutive repetitive units. The ONT reads also permitted the determination of genomic localization of rDNAs in the *C. briggsae* genome. We observed strain-specific composition and CNV of the 5S rDNA units that are consistent with the separation time among *C. elegans* strains. Our functional characterization of the 5S rDNA cluster indicates that the genomic environment of the 5S rDNA cluster is transcriptionally compatible with RNA polymerase II at least in the somatic tissues. Our structural and functional characterizations of the 5S rDNA clusters lay a foundation for study of rDNA function, regulation and evolution.

## Results

### Genomic architecture of the 5S rDNA cluster

**Fig. 1:**
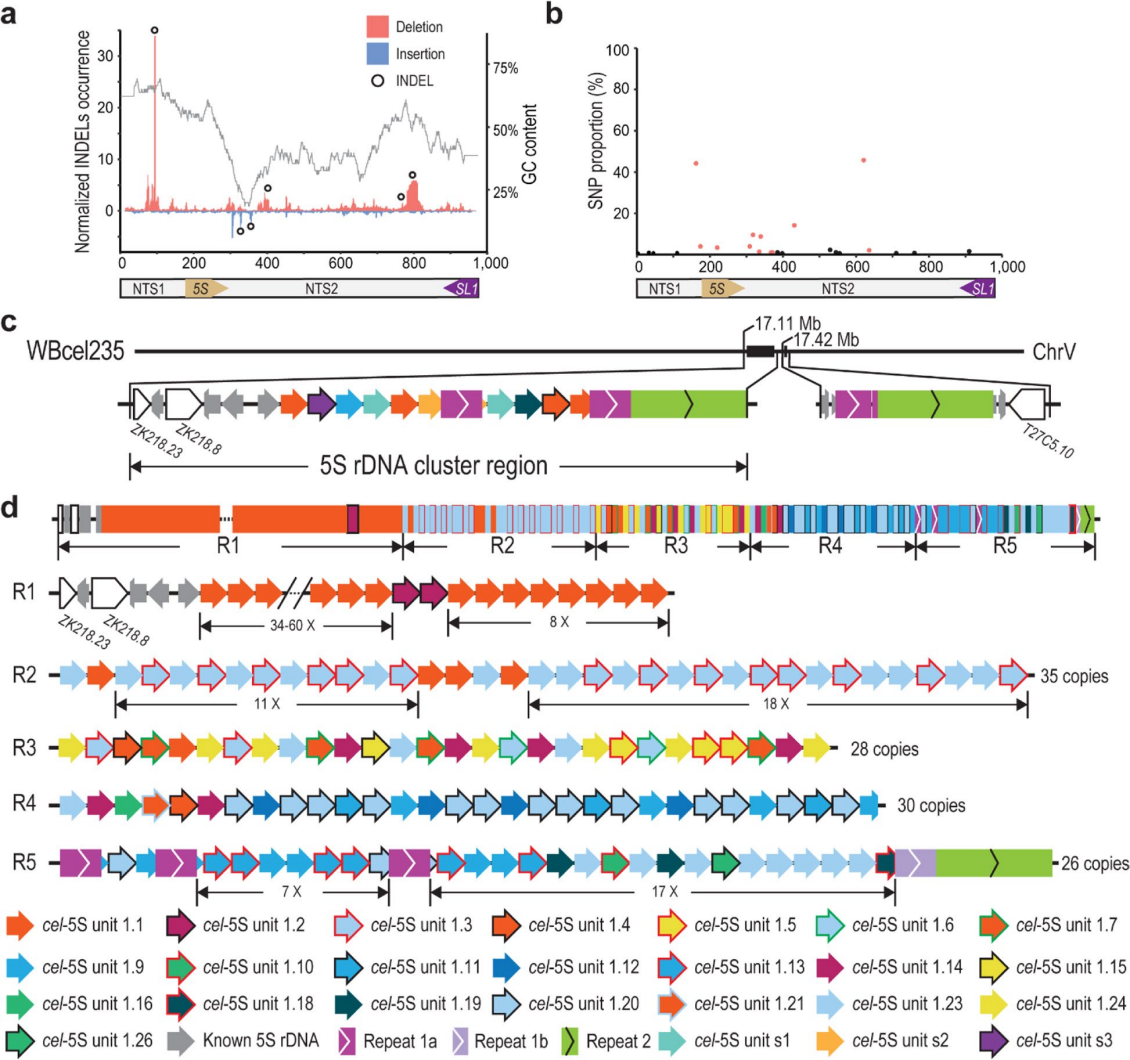
Structure of the *C. elegans* (N2a) 5S rDNA cluster. **a** INDELs identified with Nanopore reads within the 5S rDNA unit. Shown are normalized INDEL occurrences along with GC content. Deletion and insertion identified with Nanopore raw reads are shown in red and blue, respectively. Cross-validated INDELs used in the subsequent analysis are indicated with black circles (see Methods). Two large indels are indicated. **b** SNPs in the 5S rDNA are identified with existing NGS data. SNPs present or absent in new rDNA variants are colored in red and black, respectively. **c** Structure of the 5S rDNA-containing regions on the chromosome V in the current *C. elegans* N2 reference genome (WBcel235). **d** Structure of the 5S rDNA cluster assembled by ONT reads, which carries a total of at least 167 partial or complete units. The cluster is divided into 5 regions (R1-5) based on the SNPs and INDELs present in each unit or the position relative to Repeat 1a. Newly identified rDNA units or unique repeats are differentially color coded (see Table 2). Variation in rDNA copy number is indicated with a dash line. Note that three copies of Repeat 1a are inserted into the 5S rDNA unit at the same position within R5

To gain an initial idea of the genomic architecture of 5S rDNA cluster, starting from the existing *C. elegans* N2 genome assembly WBcel235 [25], we set out to generate the assembly of 5S rDNA cluster located on the chromosome V because the 5S rDNA has a relatively small size and well-characterized boundary sequences (Fig. S1). We generated ~ 1.8 million ONT reads with an N50 from 18 to 31 Kbp from three developmental stages of *C. elegans* N2a, i.e. embryo (EMB), L1 larvae (L1), and young adult (YA) stages (Table 1), which were mapped against the *C. elegans* reference genome WBcel235 [42]. As expected, the mapping results showed a drastic increase in the read coverage of 5S rDNA compared with its flanking sequences (Fig. S2), which allowed a more reliable estimation of rDNA copy number (Table S1). The flanking sequences of the 5S rDNA cluster were identified using the ONT reads that spanned at least one rDNA gene and the unique sequences on both sides of the cluster (Fig. 1c). A recent study investigated the structure of 5S rDNA units in the Aquatic Plant *Landoltia punctata* (Lemnaceae) by PCR amplification of 5S rDNA followed by Sanger DNA sequencing [43]. No length variation was detected in the 5S rDNA gene sequence, whereas the nontranscribed spacer was found to vary from 151 to 524 bp. However, due to the relatively short read length if their sequencing reads, it is not feasible to assemble the 5S rDNA cluster. In addition, the PCR amplification step makes it difficult to estimate the copy number of rDNA. Given that the genomic structure of 5S rDNA cluster has not been resolved in other species due to its extremely repetitive features, we set out to investigate whether there were any sequence variants in the 5S rDNA units that could be harnessed to assemble the entire cluster by sequencing of three developmental stages of *C. elegans* using ONT. Unexpectedly, we not only confirmed the presence of the canonical 5S rDNA unit (referred to as unit 1.1 hereafter) in the current *C. elegans* genome (WBcel235), but also identified numerous novel variants of the 5S rDNA unit that are reproducibly arranged relative to one another in the ONT reads. We used all the variants of 5S rDNA unit and non-rDNA local repeats that were supported by at least 20 ONT reads for both strands to facilitate our assembly of the 5S rDNA cluster (Fig. 1a, b, and Table 2). We classified the remaining *C. elegans* 5S rDNA units into a total of 21 variants based on their sequence divergence from the canonical 5S rDNA unit 1.1. The relative proportion of each 5S rDNA variant with unique SNP/INDEL was confirmed with the NGS reads [44] (Fig. 1b). The units with confirmed SNPs were used to build a phylogenetic tree (Fig. S3).

**Table 1:** Read statistics

Library name	Total number of reads	Total bases (Gbp)	Mean length (bp)	Median length (bp)	N50 length (bp)	Max mapped length (bp)
**N2a-EMB**	789,871	10.8	13,724	13,084	18,558	163,153
**N2a-L1**	199,712	3.7	18,300	13,605	31,265	196,902
**N2a-YA**	822,902	9.3	11,341	9187	19,566	174,664
**AF16-YA**	1,433,280	11.1	7724	4148	15,427	182,506
**ZZY0600**	870,874	12.6	14,479	10,248	25,074	247,180
**ZZY0603**	2,696,939	12.9	4785	2463	9429	252,751
**ZZY0653**	60,187	0.6	10,720	2409	27,723	139,839
**CB4856**	2,294,403	15.1	6562	2347	19,197	382,430

*EMB* Mix-staged embryos, *L1* Larval stage 1, *YA* Young adult

**Table 2:** List of the variants of 5S rDNA unit in *C. elegans (*N2a) used in this study

Variant	Size (bp)	Copy number	Sequence variation relative to *cel*-5S unit 1.1
unit 1.1	976	Dynamic	Not applicable
unit 1.2	971	2	766_771delinsC
unit 1.3	972	16	99_102del, 162C > G
unit 1.4	976	2	621 T > G
unit 1.5	946	3	780_809del, 621 T > G
unit 1.6	972	2	99_102del, 162C > G, 220C > A, 621 T > G
unit 1.7	976	4	220C > A, 621 T > G
unit 1.9	976	9	99_102del, 162C > G, 318 T > C, 325_326insCAAT, 329G > T, 332 T > G, 339C > T, 621 T > G
unit 1.10	976	1	99_102del, 162C > G, 318 T > C, 325_326insCAAT, 329G > A, 332 T > G, 339C > T, 621 T > G
unit 1.11	976	3	99_102del, 162C > G, 318 T > C, 325_326insCAAT, 329G > T, 332 T > G, 339C > T, 431 T > G, 621 T > G
unit 1.12	963	4	99_102del, 162C > G, 318 T > C, 325_326insCAAT, 329G > T, 332 T > G, 339C > T, 393_405del, 431 T > G, 621 T > G
unit 1.13	976	6	99_102del, 162C > G, 318 T > C, 325_326insCAAT, 329G > A, 332 T > G, 339C > T, 431 T > G, 621 T > G, 636 T > G
unit 1.14	980	6	309 T > C, 318 T > C, 325_326insCAAT, 329G > T, 332 T > G, 621 T > G
unit 1.15	950	1	780_809del, 220C > A, 309 T > C, 318 T > C, 325_326insCAAT, 329G > T, 332 T > G, 621 T > G
unit 1.16	976	1	99_102del, 162C > G, 309 T > C, 318 T > C, 325_326insCAAT, 329G > T, 332 T > G, 621 T > G
unit 1.18	984	1	99_102del, 162C > G, 309 T > C, 318 T > C, 325_326insCAAT, 329G > T, 332 T > G, 354_355insGGTATT, 367A > T, 371 T > A, 621 T > G, 718_719insGA
unit 1.19	982	3	99_102del, 162C > G, 309 T > C, 318 T > C, 325_326insCAAT, 329G > T, 332 T > G, 354_355insGGTATT, 367A > T, 371 T > A, 621 T > G
unit 1.20	972	15	99_102del, 162C > G, 431 T > G, 621 T > G
unit 1.21	976	1	162C > G, 621 T > G
unit 1.23	972	29	99_102del, 162C > G, 621 T > G
unit 1.24	942	7	780_809del, 99_102del, 162C > G, 621 T > G
unit 1.26	976	1	99_102del, 162C > G, 335G > C, 407C > T, 621 T > G
unit s1^a^	975	0	99_102del, 162C > G, 318_319insA, 390delC, 621 T > G
unit s2^a^	981	0	354_355insGGTATT, 367A > T, 371 T > A, 545G > A, 621 T > G
unit s3^a^	972	0	325_326insCAAT, 329G > A, 332 T > G, 339C > T, 431 T > G,621 T > G

^a^Combinations of variants in s1-s3 are not identified in the 5S rDNA cluster. *Del* deletion, *Ins* insertion, *Delins* deletion followed by insertion

The genomic organization of rDNA units was resolved through tiling of ONT reads from both orientations by taking advantage of different combinations of rDNA unit variants and other types of repeat or transgenes present in the proximity of rDNA units within the 5S rDNA cluster (Fig. 1a-c, Fig. S1, Table 2, and Tables S2-3). Consequently, we were able to generate a contig that carries a total of at least 167 copies of 5S rDNA units (Fig. 1d), including at least 47 copies of canonical rDNA unit (unit 1.1), 116 copies of unit variants, and 4 copies of existing 5S rDNA unit 1.1. In addition, there are 3 copies of existing non-rDNA repeat (referred to as Repeat 1a, 1b, and 2) (Table S4) in the cluster. To facilitate our description, the 5S rDNA cluster was divided into five regions (R1-5) based on the number and composition of the 5S rDNA units. The results show that the 5S rDNA cluster consists of various unit variants arranged in a reproducible order in the N2a strain. Availability of the detailed structure of the 5S rDNA cluster is expected to facilitate functional and evolutionary study of 5S rDNA genes.

### Structural variations of 5S rDNA cluster between N2a and its derived *C. elegans* strains

**Fig. 2:**
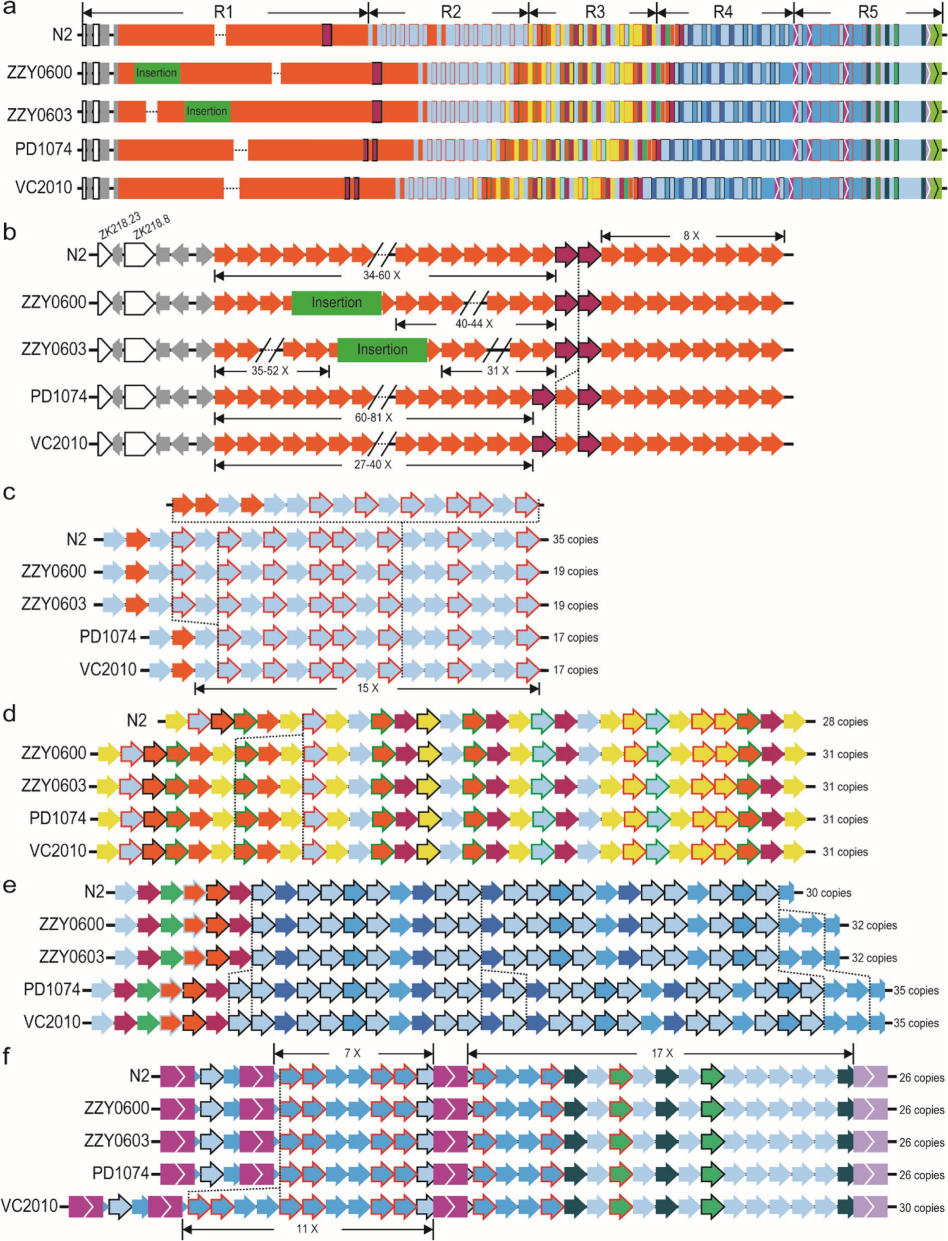
Structural variations within the 5S rDNA cluster between our *C. elegans* N2a and other N2-derived strains. **a** Overview of the structures of 5S rDNA clusters for five strains as shown in Fig. 1d. Strain names are indicated on the left. Position and size of transgenic insertions are indicated in scale. **b** Comparison of unit compositions and estimated copy number in R1. Identified variation in unit composition is highlighted with a vertical dashed line. **c**-**f** Comparison of unit compositions in R2 (**c**), R3 (**d**), R4 (**e**), and R5 (**f**) as in (**b**). **g** Ancestry of the strains based on strain history. Our N2a was shipped from Waterston lab in 2010. PD1074 was a recent derivative of VC2010 that was derived from a separate N2 in Don Moerman lab. ZZY0600 and ZZY0603 were generated by transgene insertion into *unc-119*(*tm4063*) worms, which was derived from another *C. elegans* N2 in Mitani lab

Given the relatively stable copy number and genomic organization of 5S rDNA variants in the ONT reads derived from the *C. elegans* N2a, we wondered to what extent such arrangement and copy number are conserved between the N2a and other N2-derived strains that had been separated from one another for different times. To this end, we generated ~ 0.9 and ~ 2.7 million ONT reads for two transgenic strains (ZZY0600 and ZZY0603), each carrying a single copy of transgene associated with 5S rDNA sequences (Fig. 2a, b) generated using *miniMos* technique [45] in the background of the *unc-19* mutant allele *tm4063* [46]. The reads with transgene sequences would help us to assemble the 5S rDNA cluster. However we still failed to find any such reads that could bridge the gap in the assembled 5S rDNA cluster (Fig. 1d).

Nevertheless, we found three rDNA structural variations between the 5S rDNA clusters of the N2a and the two transgenic strains (Fig. 2c-e). In addition to our sequenced data from N2a and the transgenic strains, we also used the existing ONT reads generated from other N2-derived strains [34, 36] to further evaluate the variation in the 5S rDNA clusters because the two N2 strains were separated from each other for at least 10 years. Intriguingly, we observed variations across the Region 1-4. The extent of variation is consistent with the separation time between each other, i.e., the longer time the two strains were separated from each other, the more variations were found between the structures of their 5S rDNA clusters. For example, a fragment consisting of each one copy of unit 1.1, 1.7, and 1.24 is missing in the Region 3 of our N2a relative to all the remaining strains (Fig. 2d).

More variations in the copy number of *C. elegans* 5S unit (*cel*-5S unit) 1 were observed in the Region 4 (Fig. 2e). For instance, our N2a contains 30 copies of 5S rDNA unit, whereas the two transgenic strains derived from the same starting strain contain 32 copies, and the strain VC2010 and its recent derivative PD1074 both carry 35 copies. However, the VC2010 [34] gains an extra four copies of 5S rDNA unit after its separation from its derived strain PD1074 (Fig. 2f) [38]. This apparent association of rDNA type and/or exact copy number with separation time raises the possibility of using the variation in barcoding the strains that have been recently separated from one another.

### Largely uniform composition of 5S rDNA unit in the 5S rDNA cluster of *C. elegans* Hawaii strain and *C. briggsae* wild isolate AF16

**Fig. 3:**
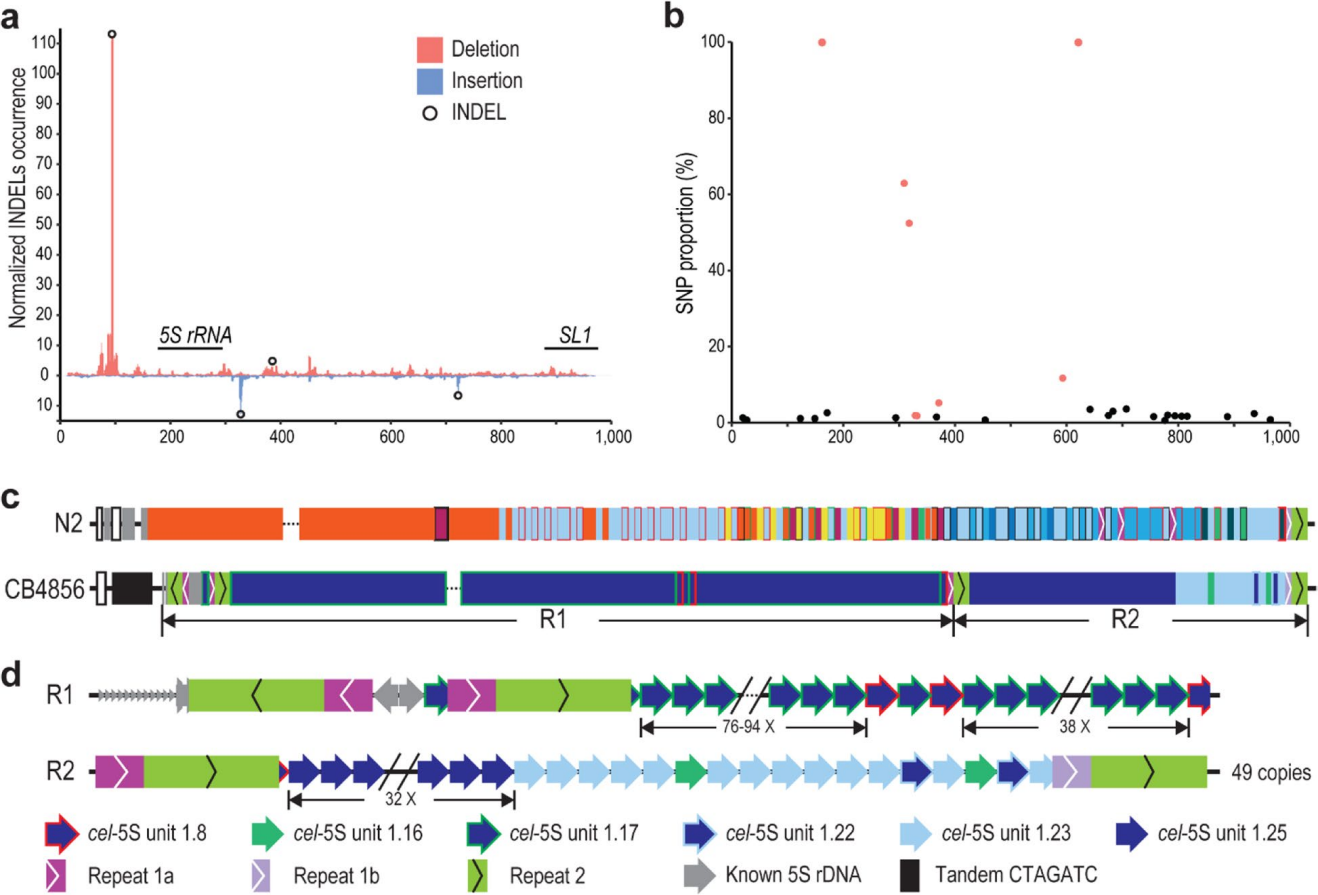
Structural variations within 5S rDNA clusters between *C. elegans* N2a and CB4856 strains. **a** INDELs identified with CB4856 ONT reads within the 5S rDNA unit as in Fig. 1a. Cross-validated INDELs used in the subsequent analysis are indicated with black circles (see Methods). **b** SNPs in the 5S rDNA are identified with existing NGS data as in Fig. 1b. SNPs present or absent in new rDNA variants are colored in red and black, respectively. **c** Overview of the structures of 5S rDNA clusters between N2a and CB4856 as shown in Fig. 1d. Note the differences between the two, including lack of unit 1.1 (red) predominantly seen in N2a, whereas the unit (*cel*-5S unit 1.17 (see Table 2 & S5)) is unique to and predominantly seen in CB4856. **d** Structure of the *C. elegans* CB4856 5S rDNA cluster. The Repeat 1a and 1b are shown as in Fig. 1d

To further examine the structural variations in 5S rDNA cluster between N2a and more distantly related *C. elegans* strains, we focused on the comparison between N2a and CB4856, a Hawaiian *C. elegans* strain that is one of the most divergent from the strain N2 [47]. To this end, we generated ~ 2.3 million ONT reads using CB4856 animals (Table 1), which were used to assemble the 5S rDNA cluster of CB4856 in a way similar to that used for the N2a (Fig. 3). Surprisingly, we found that the canonical *C. elegans* 5S rDNA unit, i.e., *cel*-5S unit 1.1, one of the most predominant forms in N2a, and many other types of variants were absent in the CB4856 genome using a combination of existing NGS reads with our ONT reads for CB4856 (Fig. 3a-d, Table 1, and Table S5). Remarkably, the occurrences of SNP and INDEL identified in the N2a 5S rDNA unit are much lower in the CB4856 than in the N2a strain (Fig. 3a-b). All the units in CB4856 carry a 4 bp-deletion (Fig. 3a and Table S5). They can be further divided into six variants versus the 26 in the N2a (Table 2). Only two out of the six variants, i.e., unit 1.16 and 1.23, are shared between the two strains. Notably, the entire 5S rDNA cluster is primarily comprised of two CB4856 unique rDNA variants, i.e., the unit 1.17 and 1.25, with the former as the predominant member (Fig. 3c-d). The relatively uniform composition of rDNA units in CB4856 is in sharp contrast to the mosaic compositions of rDNA units in the N2a (Fig. 3c). The two CB4856-specific 5S rDNA variants 1.17 and 1.8 were interrupted by the Repeats 1a and 2 at the same unit position (947-953 bp) as the variant 1.18 in the N2 (Figs. 1d and 3d), raising the possibility of their common origin.

Given the presence of a 30-bp deletion in the 5S rDNA in N2a but not in CB4856 (Figs. 1, 2 and 3, Table 2 and Table S5), we evaluated the distribution dynamics of the deletion using the existing NGS data from 330 *C. elegans* wild isolates [44]. The result confirmed the presence of the 30-bp deletion in 164 strains (including N2a) but not in the remaining strains (including CB4856) (Fig. S4, Table S6). It also showed that this unique deletion had undergone multiple times of gain or loss between strains, suggesting a high turnover rate of the deletion.

**Fig. 4:**
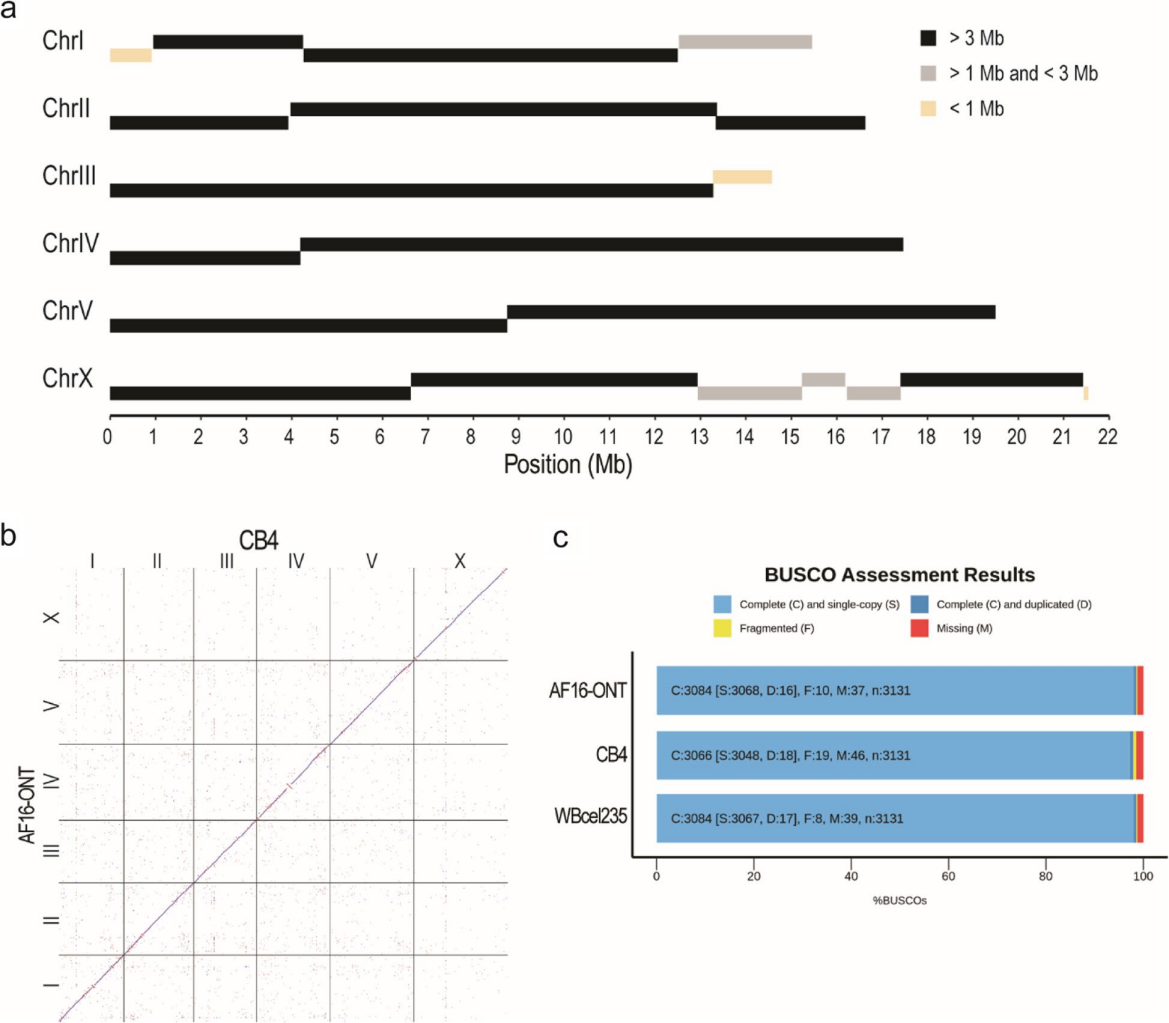
Evaluation of the ONT reads assembled *C. briggsae* AF16 genome. **a** Schematic representation of AF16 long reads assembled contig lengths. **b** Dot plot of corresponding chromosomes between CB4 and ONT reads assembled genome. **c** Bar chart with summary assessment for the proportion of genes present in three assembled genomes. AF16-ONT: the assembled *C. briggsae* draft genome in this study, WBcel235: the *C. elegans* N2 reference genome, CB4: the *C. briggsae* AF16 reference genome

**Fig. 5:**
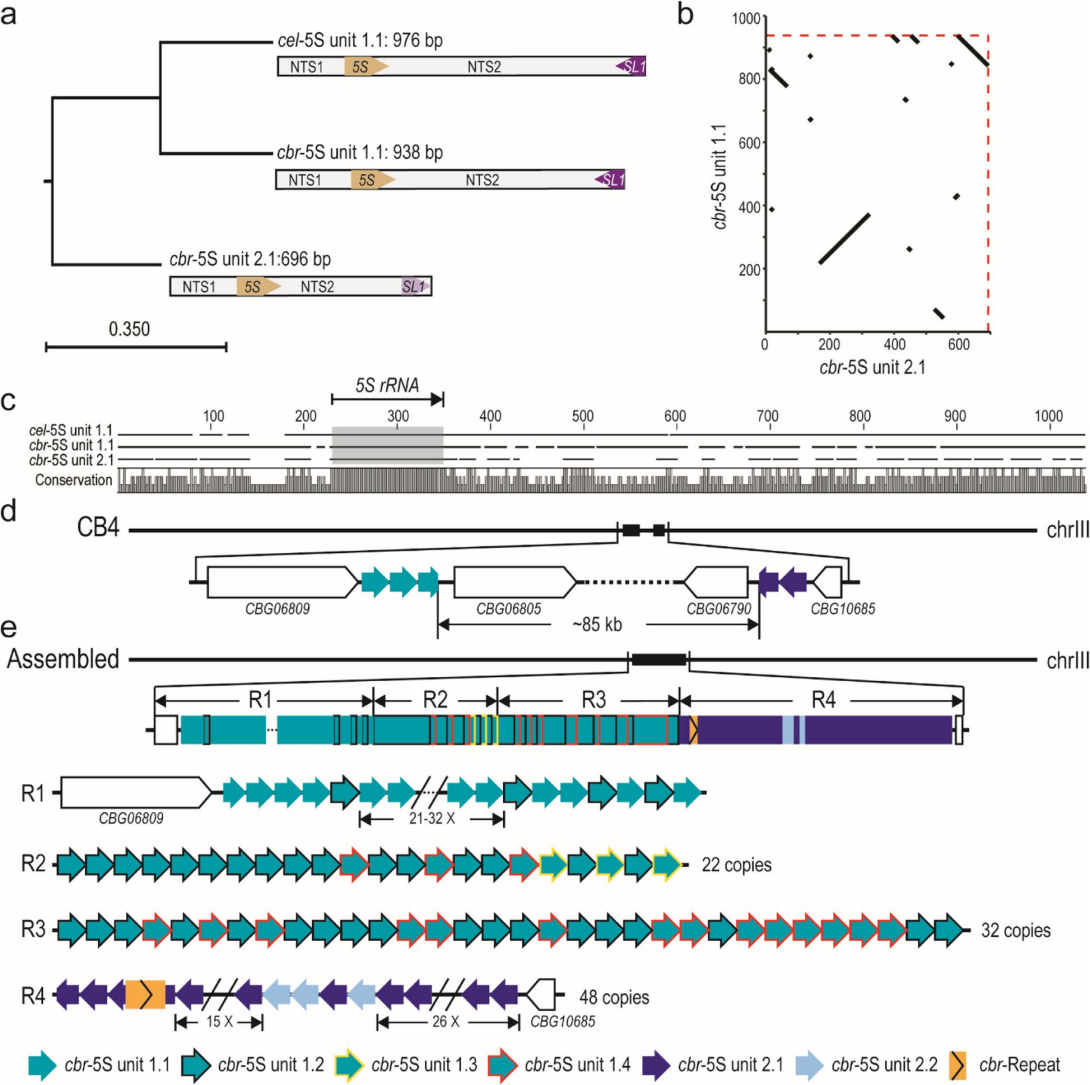
Characterization of the 5S rDNA units in *C. briggsae* AF16. **a** Phylogenetic tree of two divergent 5S rDNA units in *C. briggsae* (*cbr*) and the canonical *C. elegans* (*cel*) 5S rDNA unit. **b** Dot plot showing the sequence alignment between two *C. briggsae* 5S rDNA units. **c** Multiple sequence alignment of 5S rDNA units from *C. elegans* and *C. briggsae*. Alignments for the 5S rRNA gene are shaded in the grey box (indicated at the top). **d** A contig was misassembled into the rDNA cluster on chromosome III in the reference genome CB4. **e** Schematic representation of *C. briggsae* AF16 5S rDNA cluster annotated by ONT reads

To further examine to what extent the structure of the 5S rDNA cluster is conserved between species, we generated ~ 1.4 million of ONT reads (approximately 91× coverage) using genomic DNAs from *C. briggsae* AF16 young adults with an N50 of ~ 15.4 kb and ~ 39 million of paired end NGS reads of 150 bps in length from mix-staged *C. briggsae* animals. To locate the flanking sequences of 5S rDNA cluster in the *C. briggsae* genome, we combined the ONT reads with the previous SLR reads [27] to generate an AF16 genome assembly using Miniasm [48], followed by polishing with Racon [49]. After removal of bacterial genome and duplicated contigs, this draft assembly contains 20 contigs with summed size of approximately104 Mbp (Fig. 4a). The contigs were ordered and oriented relative to one another with the reference to CB4 [28] (Fig. 4b). Evaluation using BUSCO [50] revealed the completeness of this genome assembly was comparable to that of the *C. elegans* N2 genome (Fig. 4c). The *C. briggsae* genome was known to contain two divergent 5S rDNA units with an opposite orientation of SL1 relative to 5S rDNA gene sequence. They were referred to as *cbr*-5S unit 1.1 and 2.1, respectively (Fig. 5a-c), which were previously placed onto two separate locations of different chromosome [32] (Fig. 5d). With two SNPs in *C. briggsae* 5S unit (*cbr*-5S unit) 1.1 (195G > T and 674G > T) and one deletion identified in the NGS data relative to *cbr*-5S unit 1.1 and 2.1 (382_440del), respectively (Table S7), we classified the *C. briggsae* 5S units into six types, i.e., unit 1.1-1.4 and unit 2.1-2.2, and generated the 5S rDNA cluster assembly in *C. briggsae* (AF16) in a similar way to our work in *C. elegans*. Our new genome assembly and existing Hi-C data [32] supported that all the six divergent 5S rDNA units were located within a single location in the *C. briggsae* genome (Fig. 5e and Fig. S5b). The results also showed that the *C. briggsae* 5S rDNA cluster mainly consisted of four types of unit, i.e., 1.1, 1.2 1.4 and 2.1 (Fig. 5e). In summary, although the variations in sequence and copy number of 5S rDNA unit are quite common in *C. elegans* N2 and its derived strains, the 5S rDNA unit is largely uniform in *C. elegans* Hawaii strain (CB4856) and *C. briggsae* wild isolate (AF16), suggesting that the N2 may have a defective system in maintaining the stability of its 5S rDNA cluster.

### Transposition of chromosome I end associated with 45S (18S-5.8S-26S) rDNA cluster in the *C. elegans* genome

**Fig. 6:**
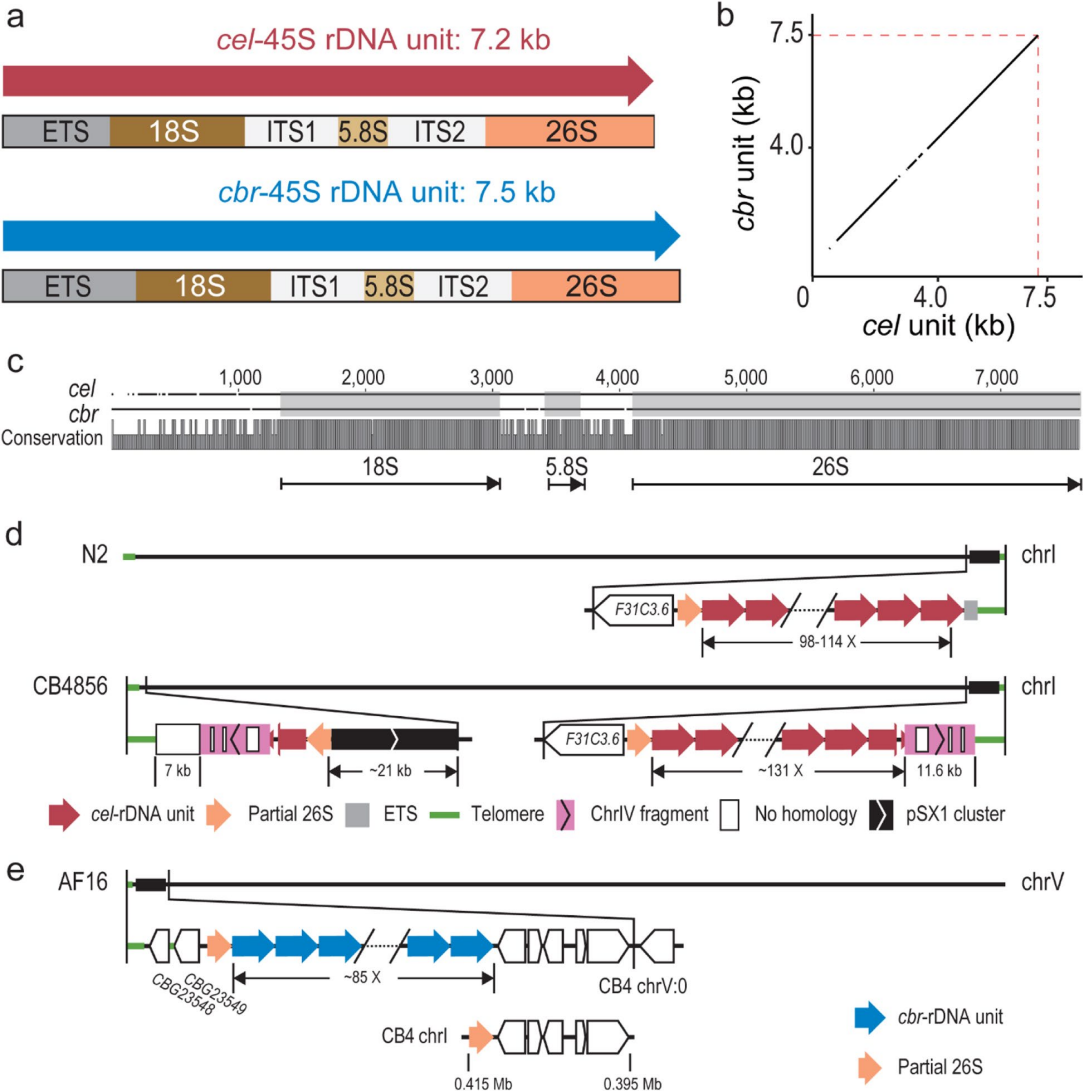
Comparison of 45S rDNA units and clusters between strains and species. **a** Comparison of 45S rDNA units between *C. elegans* and *C. briggsae*. **b** Dot plot showing the alignment of the 45S rDNA unit sequences between two species. **c** Pairwise sequence alignment of the 45S rDNA unit between two species. The 18S, 5.8S, and 26S RNA gene regions are shaded in grey. Conservation scores are shown at the bottom. **d** Schematics of the 45S rDNA cluster of *C. elegans* N2a and CB4856 annotated by ONT reads. In the N2a, the cluster left and right boundaries are flanked by partial 26S rRNA sequences and a partial ETS, respectively. In the 45S rDNA-containing region in *C. elegans* CB4856, the 45S rDNA cluster is located at the right end of chromosome I while fragmented 45S rDNA sequences along with other sequences are located at the left end. The estimated copy number of the unit is shown. Note that both the chromosome left and right ends are flanked by a ~ 11.6 kb fragment derived from the left end of chromosome IV (pink, see Fig. S8), which is interrupted by some no homologous sequences (white box). A pSX1 cluster is also found adjacent to 45S rDNA. **e** Schematics of the *C. briggsae* AF16 genomic regions containing the 45S rDNA annotated by ONT reads in this study. Reconstructed 45S rDNA cluster is located at the left end of chromosome V containing about 85 copies of the 45 rDNA unit. Bottom: A misassembled contig containing partial 26S rRNA gene sequences and 5 protein coding genes was assigned to chromosome I in CB4

The 5S rDNA cluster is separated from the 18S-5.8S-26/28S rDNA cluster in nematodes [2, 21]. The 45S rDNA unit consists of an 18S, a 5.8S and a 26S rRNA gene interrupted by two internal transcribed spacers (ITS1 and ITS2) in both *C. elegans* and *C. briggsae* (Fig. 6a). The *C. briggsae* 45S rDNA unit is roughly 300 bp longer than that of *C. elegans*, which was mainly contributed by the external transcribed sequence (ETS) (Fig. 6a-c). The *C. elegans* 45S rDNA cluster is located at the right end of chromosome I. The ONT reads from all *C. elegans* N2a-derived strains confirmed that the sequence between the 45S rDNA cluster and the telomere sequences is partial ETS (Fig. 6d). Based on the NGS reads of N2 genomic DNAs [44], most of the called variants using ONT reads (Fig. S6 and Table S8) resulted from INDELs in the homopolymer regions, in which ONT read sequences were known to be less reliable, and our attempt to identify possible sequence variation within the cluster was unsuccessful. In addition, all our ONT reads carrying either the left or the right flanking sequences contain only partial 45S rDNA unit. This was mostly due to the relatively large size of the unit (~ 7.2 kb in *C. elegans* and ~ 7.5 kb in *C. briggsae*) and a relatively shorter 45S rDNA sequence-containing reads compared to other genomic positions (Fig. S7). Therefore, we were unable to identify any unique sequence in the cluster as an anchor to extend ONT reads deeper into the cluster from both boundaries. Although we were not certain whether there were any structural variations within the *C. elegans* 45S rDNA cluster, these ONT reads can be used to correct the boundary sequences of the 45S rDNA cluster in *C. elegans* N2a and CB4856 strains (Fig. 6d). We observed a dramatic rearrangement event in the right boundary of CB4856 chromosome I relative to that of N2a. For example, we identified an apparent transposition of the left end of chromosome IV to the right end of chromosome I of CB4856 genome (Fig. S8), which is consistent with a previous finding [39]. The transposed fragment underwent a duplication and transposition to the left end of the chromosome I along with its flanking rDNA sequences. A tandem array consisting of positioning sequence on X (pSX1) [51] was also found adjacent to the transposition site, but its origin was unclear.

In the *C. briggsae* genome assembly CB4 [28], the 45S rDNA-containing sequences were fragmented in various contigs with unknown chromosome linkage (Fig. 6e and Fig. S2d). The Hi-C data [32] and our ONT reads supported a single location of the 45S rDNA cluster at the left end of the chromosome V (Fig. 6e and Fig. S4b). We further evaluated the validity of the estimated copy number of the 45S rDNA unit by mapping our ONT reads against the 45S rDNA cluster consensus sequences incorporated into our newly generated *C. briggsae* genome. The changes in reads coverage were consistent with the estimation of 45S rDNA copy number (Fig. S2d).

### The genomic environment of rDNA cluster is compatible with RNA pol II transcriptionally

Eukaryotic cells use at least three RNA polymerases, i.e., RNA polymerase I (Pol I), Pol II, and Pol III, which produce 18S/5.8S/26(28)S rRNAs, mRNAs, and 5S rRNAs, respectively. Given that all the rDNAs transcribed by the RNA Pol I and III are localized at two distinct loci consisting of rDNA and some other repetitive sequences only but depleted of any protein-coding sequences in both the *C. elegans* and *C. briggsae* genomes, and a yeast mutant lacking rDNA locus can be rescued by forced expression of rRNAs by RNA polymerase [52], we wondered whether the two rDNA clusters are permissive to RNA Pol II transcriptionally in nematode as in yeast [6]. To this end, we generated multiple transgenic lines carrying a single copy of insertion within or outside the rDNA cluster expressing a fluorescence marker along with a copy of *unc-119* gene [46]. In the transgenic animals, a complete rescue of the uncoordinated phenotype along with expression of the reporter in some parts of the soma indicates the native rDNA cluster regions are transcriptionally compatible with Pol II in the somatic tissues (Fig. S9), consistent with the observation in the yeast [6]. However, despite the expression of the reporter in soma, germline, and early embryo when it was inserted outside of the rDNA cluster, the expression in germline and early embryo was absent for the same reporter inserted within the rDNA cluster, suggesting that the genomic environment of rDNA cluster may not be accommodative to the expression in the germline and early embryo. It is worth noting that only a single promoter reporter fusion was tested in this case, and the integrity of the transgene was not examined by sequencing except for its insertion site. This difference in expression could reflect a positional effect or is unique in *Caenorhabditis* species. Studies in *Drosophila* show that insertion of a heterologous sequences into rDNA leads to decreased expression [53]. Impaired function of rDNA transcription initiation machinery leads to derepression of ribosomal genes with insertions of R2 retrotransposon [54]. Studies in *Arabidopsis* reveals that different 5S rDNA units are subjected to differential epigenetic regulation and prone to translocation between strains [55]. More robust tests are needed with multiple independent fusion reporters to validate whether the observed differences in the expression patterns are really caused by the insertions inside or outside of the rDNA cluster.

## Discussion

Rapid development in sequencing technologies that can produce ultra-long reads makes it possible for resolving the structures of complex genome regions, including those consisting of tandem repetitive sequences. These sequences represent the “dark matter” of the existing genomes, including the human genome [11]. One of the key advantages of the long reads is their ability to span repetitive sequences, allowing de novo assembling of the repetitive region or scaffolding of the existing contigs generated from NGS reads. Aided by the long reads, it becomes within reach to resolve the structure of highly repetitive regions, including rDNA cluster, centromere, telomere, or chromosomal rearrangement. Our analyses of rDNA cluster structures using ONT long reads in both *C. elegans* and *C. briggsae* provide insights into the intra- or inter-species dynamics of rDNA clusters, which demonstrate an unusual high rate of structural and sequence variations inside the 5S rDNA cluster in the *C. elegans* N2 strain compared with its distantly related *C. elegans* CB4856 strain and the *C. briggsae* AF16 strain. The results suggest that the *C. elegans* N2 strain is deficient in maintaining the structure and stability of its rDNA cluster relative to other strains or *Caenorhabditis* species. This may have implications for its fitness, which warrants further investigation.

### Potential biological implications of the heterogeneity of 5S rDNA sequence and copy number in *C. elegans*

It has been demonstrated that a substantial divergence in the rRNA gene sequences are present within individual microorganisms, which plays an important role in the regulation of gene expression at the ribosome level [56]. Highly abundant variations in the rRNA genes are also observed in human and mouse, and these alleles are conserved and exhibit tissue-specific expression [57]. Notably, nearly all of the INDELs identified in this study are located in the regions outside of the rDNA genes (Figs. 1a and 3a), whereas the SNPs are found in both rRNA genes and NTS 1 or 2 (Figs. 1b and 3b). Given the high level of heterogeneity in 5S rDNAs across the *C. elegans* N2 and its derived strains but not in the CB4856 strain, a few questions remain unanswered. For example, are all these 5S rDNA units transcriptionally active or are they on the way to degeneration? If they are transcriptionally active, are their transcriptions equally effective, especially for those 5S rDNA units that were interrupted by another repeat within the unit? Second, are these unit variants differentially used in a tissue- or developmental stage-specific manner as seen in other species if they are functional? Third, what are the mechanisms for maintaining the copy number and organization of the rDNA unit variants in a given strain? Fourth, what is the biological implications of an elevated rDNA heterogeneity in the N2 versus the CB4856 strain of *C. elegans*?

The tandemly repeated nature of rDNA units creates an inherent instability for rDNA loci due to intrachromatid homologous recombination between copies [58]. The copy number variation probably reflects the effects of natural copy number loss and the recovery of copy numbers to maintain the functionality of the rDNA cluster or their combination [59]. Both genetic and environmental factors have been reported to regulate rDNA copy number. For example, studies in *Arabidopsis* have demonstrated that most of its rDNA copies are silenced, and rDNA silencing is mediated through DNA methylation and histone modification [60]. The defects in silencing lead to aberrant rDNA copy number [61]v. Copy number loss may occur during aging. For example, in budding yeast, mother cells age progressively with each division and eventually die after ~ 20 cell divisions due to rDNA instability caused by intrachromatid recombination that reduces chromosomal rDNA copy number and generates extrachromosomal rDNA circles (ERCs) [62]. However, evidences show there must be some mechanisms in place to restore rDNA copies and counteract the loss over generations. Study in budding yeast suggest a process called unequal sister chromatid recombination (USCR), in which strand invasion can occur in a way that increases rDNA copies upon completion of repair [63]. In addition to USCR, reintegration of ERCs has been proposed as a potential mechanism to increase chromosomal rDNA copy number [64].

Given that the composition of 5S rDNA unit variants in the N2 and its derived strains is relatively stable across generations, it is expected that the regions rich in these variants are not undergoing active homologous recombination through unequal crossover. Otherwise, a highly homogeneous configuration of 5S rDNA unit is expected [65]. A slight variation in the copy number of the 5S rDNA unit among N2 and its derived strains appears to be a product of genetic drift. This is because these strains have not been under obvious selection pressures except for having been separated from one another and maintained at different labs for different time. Further analysis is needed to unravel the biological significances of these variants during development or under environmental stimuli.

### The power of ONT reads in resolving tandem repeats

Repetitive sequences, especially those tandem repetitive ones, are problematic for genome assembly. The *C. elegans* genome has been claimed as a “finished” genome with no gap due to its homozygosity and relatively small size [25]. However, the annotation of its genomic regions involving rDNA sequences is far from completion. For example, except for the boundary sequences, the previous sequencing methods failed to establish the genomic arrangement of the rDNA units and their variations [25, 34, 38]. Meanwhile, the existing *C. briggsae* genome assembly is far more fragmented than the *C. elegans* one. Despite multiple attempts to improve the genome assembly of *C. briggsae* [23, 27, 28, 32, 66], the structure and genomic localization of rDNA clusters has not been resolved. Aided by the ONT reads of high coverage, the genomic localization was readily resolved for both 5S rDNA and 45S rDNA clusters in *C. briggsae* (Fig. 5e and Fig. 6e). Our method of using ONT sequencing in resolving complex genomic structures and repetitive regions is also applicable to rDNA clusters in other species. For example, taking advantage of ONT reads, the entire human X and Y chromosomes were assembled from telomere to telomere using genomic DNAs of an isogenic cell line [67, 68].

Most existing nematode genomes were assembled as contigs using shotgun sequencing method with NGS reads [69], which is also the case for many other species, leading to the genomic gaps consisting of tandem repetitive sequences. Due to the decreasing sequencing costs using ONT or other TGS platforms, it is feasible to re-sequence or improve the existing genomes especially for those of human and model organisms as well as economically significant species using the reads produced by ONT or other sequencing platforms such as PacBio High-Fidelity (HiFi). For example, a new genome assembly has been recently produced for human and vertebrates with a combination of HiF and ONT reads using isogenic genome from a cell line [70]. Notably, the human genome assembly generated with HiFi reads does possess a low level of heterozygosity, including a megabase-scale heterozygous deletion within the rDNA array on Chromosome 15, which was revealed by ONT sequencing, highlighting the role of ONT sequencing in resolving regions containing highly tandem repeats. The ultra-long ONT sequencing excels at spanning long, identical repeats, whereas HiFi sequencing excels at differentiating subtly diverged repeat copies or haplotypes [70]. Using long ONT reads, structural variations including duplication and inversion can be easily picked up. However, the differences at base level could be missed by the poor accuracy of base-calling of ONT reads. Especially, the strand-specific errors have been observed in the earlier investigations, which could lead to mis-assembly of a genomic region using different algorithms [71]. Given a relatively lower read accuracy of ONT reads than NGS reads, it would be ideal to simultaneously generate new or use the existing NGS reads to correct the nucleotides of a de novo genome assembly generated with ONT reads only. This would give rise to a highly accurate genome in terms of nucleotide and chromosome continuity.

### Failure of recovering any ONT read that spans the entire 5S rDNA cluster suggests a complex structure of the cluster

Given the large ONT read size of up to 196 Kbps (Table 1), the estimated copy number (Table S1), and relatively small size of the 5S rDNA unit, we expected that there were at least some ONT reads that were able to span the entire region from the left boundary of the 5S rDNA cluster to the “anchoring” sequence, i.e., the unique variants of 5S rDNA unit or the transgenes landed inside the cluster (Fig. 2a, b). However, we failed to recover any of such ONT reads, suggesting that there could be some complex structural barriers that somehow prevented the sampling of full-length DNAs that spanned the entire cluster, especially the region with homogenous unit composition. In addition, we observed a relatively smaller average read length of ONT reads associated with rDNAs than those independent of rDNAs (Fig. S7). For example, in the strain ZZY0603, which carries a transgene inside the 5S rDNA cluster (Fig. 2b), the ONT reads associated with the transgene contained up to 52 copies of the canonical 5S rDNA unit on the left side of the transgene. However, no read was found to span the entire region from the left boundary of the 5S rDNA cluster to the transgene. This was unexpected because the entire unresolved part within the R1 region was estimated to carry a total of 34-60 copies of 5S rDNA unit with 31 copies located on the right side of transgene (Fig. 2b). Similarly, in the ONT reads of ZZY0600, which carried a transgene next to the sixth copy of the 5S rDNA unit away from the left boundary of the 5S rDNA cluster, the ONT reads associated with the transgene carried a maximum of 44 copies of 5S rDNA unit on the right of the transgene (Fig. 2b). Again, no read was found to span the entire region from the anchoring 5S rDNA variant (unit 1.2) to the transgene. Therefore, we postulate that part of the rDNAs in this region may undergo active replication or transcription, which prevents sampling of a longer DNA fragment for sequencing. For example, at replication fork, the rDNAs undergoing active replication are single-stranded [64], which would be vulnerable to DNA shearing during DNA extraction, leading to the absence of the long reads spanning the entire active region. Alternatively, the failure of ONT read to span the entire region could have been caused by a complex tertiary structure of the highly repetitive DNA sequences, which might be difficult to be opened up by the helicase during ONT sequencing, leading to blocking of the flow-cell pores and thus the early termination of ONT sequencing process.

### Uncoupled 5S rDNA and 45S rDNA copy number between developmental stages at the organism level

The copy numbers among the 5S, 5.8S, and 28S rRNA genes, which encode rRNAs that constitute the ribosomal large subunit, were thought to be highly correlated [72, 73]. Given the differential transcriptional efficiencies between cell types and the storage of 5S rRNA in ribosome-free particles [74], the copy numbers of rRNA genes may not necessarily show concerted change at organism level although they could be coupled in a particular cell type. For example, the estimated copy numbers appeared to be uncoupled between 5S rDNAs and 45S rDNAs (Tables S9-10). The copy number of the 5S rDNA unit is 116, 169 and 184 in the embryo (EMB) or L1 s and young adult (YA) stage, whereas the copy number of 45S rDNA unit reached the highest level at the L1 stage (114 copies) compared with 98 and 103 copies at the EMB and YA stages, respectively. Although this result is consistent with a previous finding with mutated *C. elegans* NGS data [12], it is inconsistent with the data from human and mouse [72]. Consistent with this, meta-analysis demonstrates that thousands of high-quality sequencing samples fail to show meaningful correlation between 5S and 45S ribosomal DNA arrays in humans [75]. The results suggest differential regulations of the overall dosage of 5S and 45S rDNAs between nematodes and mammals.

In summary, the availability of ultra-long reads from ONT or PacBio platforms is expected to accelerate the generation of complete genome sequences from telomere to telomere. With these ultra-long reads of a higher read accuracy, the structure of 45S rDNA and other highly repetitive regions such as centromeres and telomeres are readily to be resolved, leading to a gap-free genome, in human, model organisms and economically important species in the years to come.

## Conclusions

In summary, by taking advantage of existing or our newly generated Nanopore DNA sequencing reads and the sequence variations present within each unit, we produced genomic assemblies of 5S rDNA cluster in the *C. elegans* N2 and its derived strains, and in the *C. elegans* strain CB4856 that is distantly related to the N2 as well as in the *C. briggsae* AF16 strain. The assembled 5S rDNA clusters contain up to 167 consecutive 5S rDNA units in the N2 strain. The ordering and copy number of various rDNA units are predictive of separation time between strains. Surprisingly, we observed a drastically reduced level of variation in the unit composition in the 5S rDNA cluster in the *C. elegans* CB4856 and *C. briggsae* AF16 strains than in the *C. elegans* N2 strain, suggesting that N2, a widely used reference strain, is likely to be defective in maintaining the 5S rDNA cluster stability. The results demonstrate that rDNA units are highly dynamic both within and between population(s) of the same species in terms of sequence and copy number. The detailed structure and variation of the 5S rDNA units within the rDNA cluster pave the way for functional and evolutionary studies.

## Methods

### Sequencing library preparation and ONT sequencing

The details of ONT sequencing libraries from this study and previous studies for different strains were listed in the Tables 1 & S1. For *C. elegans* wild isolates, genomic DNAs were extracted from the mix-staged embryos (EMB), early-stage larvae (L1) and young adults (YA) of N2 strain (shipped from Waterston laboratory, Seattle, WA, USA in 2010) (termed as N2a hereafter) or from the mix-staged animals of CB4856 strain. For *C. elegans* transgenic strains, genomic DNAs were extracted from the homozygous mix-staged animals with the following genotypes: ZZY0600 (*unc-119*(*tm4063*) III; *Is*[*sel-8p*::HIS-24::GFP::*pie-1* 3′ UTR, *unc-119*(+)] V), ZZY0603 (*unc-119*(*tm4063*) III; *Is*[*dsl-1p*::HIS-24::GFP::*pie-1* 3′ UTR, *unc-119*(+)] V), and ZZY0653 (*unc-119*(*tm4063*) III; *Is*[*his-72p*::mCherry::HIS-24::*pie-1* 3′ UTR, *unc-119*(+)] I), each carrying a single-copy of transgene in 5S rDNA cluster. For *C. briggsae* wild isolate, genomic DNAs were extracted from AF16 young adults. Animal synchronization was performed as described [76]. Before harvesting, the *C. elegans* and *C. briggsae* animals were maintained on plates of 1.5% nematode growth medium (NGM) seeded with *E. coli* OP50 at room temperature and in a 25 °C incubator, respectively. Genomic DNAs were extracted from animals with PureLink Genomic DNA Mini Kit (Invitrogen) using siliconized tubes and pipette tips to minimize shearing. 4 μg purified DNAs from each sample were used for library preparation using Genomic DNA by Ligation Kits SQK-LSK108 (ONT) for N2a and ZZY0653, and Ligation Kits SQK-LSK109 (ONT) for the remaining strains. Sequencing was performed on GridION X5 or MinION with R9.4.1 flow cell (FLO-106, ONT) using default parameters.

### Sequence acquisition and alignment

Base-callings were performed using Guppy (v3.1.5, ONT) using the high-accuracy configuration model. All the base-called reads from each library were pooled for analysis of read length distribution with SeqKit (v0.10.2) [77]. The reads were aligned against the *C. elegans* N2 genome assembly (WormBase WBcel235) [42] or the *C. briggsae* AF16 genome assembly (CB4) [28] with Minimap2 (v2.17) [78] using default parameters for ONT reads. Read coverage was calculated from the BAM file using SAMtools depth [79]. The ONT reads of *C. elegans* VC2010, a wild-type strain derived from N2, were downloaded from European Nucleotide Archive (ENA) with accession numbers PRJEB22098 [34]. The ONT and PacBio reads from *C. elegans* strain PD1074, a wild type strain derived from VC2010, were downloaded from Sequence Read Archive (SRA) database with accession number SRR7594463 and SRR7594465, respectively [38]. The ONT reads of VC2010 and PD1074 were used for identifying lab-specific variations in the rDNA unit and its genomic organization. The PacBio reads of *C. elegans* CB4856 were downloaded from the SRA database with accession number SRR8599837 [39].

For short NGS reads of *C. elegans* N2a and CB4856, the alignment BAM files were downloaded from *Caenorhabditis elegans* Natural Diversity Resource (CeNDR) project [44]. The *C. briggsae* SLR reads and Hi-C reads were downloaded from the SRA database with accession number SRR6384296 and SRR6384332, respectively [27, 32].

### Identification of variation in 5S rDNA units

*C. elegans* ONT reads with rDNA sequences were aligned against a single copy of *cel*-5S unit 1.1 with Minimap2. From the CIGAR strings in the generated SAM file, to minimize the INDELs resulting from base-calling errors for homopolymers and simple repeats, only the INDELs longer than 3 bp were kept for copy counting with custom scripts. After normalization with genome-wide read coverage, the normalized INDEL count higher than one copy was considered as a potential new INDEL variant. Two types of deletion were identified in N2a strain only, one carrying a 4-bp deletion and other a 30-bp deletion (Table 2). Using the strain-specific BAM files generated with NGS read alignment against the N2 reference genome produced previously [44], *C. elegans* N2a and CB4856 NGS reads mapped to the 5S rDNA region were separately extracted and then individually mapped to the sequence of a single *cel*-5S unit 1.1 in the same way as that for the ONT reads. SNP calling within rDNA unit was performed with BCFtools [80] using the NGS reads stated above.

The sequences of all identified *C. elegans* 5S rDNA units (excluding the two deletions) were used for multiple alignment and construction of phylogenetic tree with CLC Sequence Viewer (v8.0, QIAGEN) using following parameters: gap open cost: 10.0; gap extension cost: 1.0; and alignment mode: very accurate. Neighbor joining phylogenetic trees of 5S rDNA units from each species were generated with Jukes-Cantor distance measure with 1000 replicates of bootstrapping. The individual 5S rDNA unit variants was named based on their relatedness to the *cel*-5S unit 1.1 in the tree (Fig. S3).

To investigate whether the 30-bp deletion in the *C. elegans* 5S (N2a) units are present in all *C. elegans* wild isolates, the NGS reads derived from 330 whole-genome shotgun sequencing libraries [44] were mapped against the sequences of only two *C. elegans* 5S rDNA that carry the 30-bp deletion, i.e., unit 1.1 and 1.24, using BWA (v0.7.17) [81]. The reads that were uniquely mapped to the deletion junction for at least 12 bps at both flanking sides were extracted with SAMtools with parameters -q 30 -F 4. A strain was defined as the 30-bp deletion-containing if over 1% of total reads carried the deletion regardless of the total number of supporting reads, or if over 0.1% of total reads carried the deletion but with at least 10 supporting reads. The presence and absence of the 30-bp deletion on a phylogenetic tree of the 330 strains produced previously [44] was visualized in R with ggplot2 and ggtree packages [82–84]. The 5S rDNA unit variants of *C. elegans* CB4856 strain and *C. briggsae* AF16 strain were identified similarly as those in the *C. elegans* N2a strain.

### Reconstruction of rDNA clusters

Reconstruction of the *C. elegans* 5S rDNA cluster started with identifying all the ONT reads carrying the flanking sequences of the cluster, i.e. the *ZK218.23* as the left boundary, and the sequences from chrV: 17,133,740-17,137,381 (WBcel235) as the right boundary. These reads were iteratively extended into the cluster by performing SNP- and INDEL-based manual assembly. Based on the pairwise alignment results using BLASTN [85], the consensus of 5S rDNA cluster was built using at least 10 supporting ONT reads that contained the sequences of rDNA variants or other repeats as anchors from both DNA strands (Fig. S1). This step was reiterated till the exhaustion of all available ONT reads. To determine the potential structural variations among *C. elegans* N2a-derived strains and between *C. elegans* strains, each 5S rDNA cluster was similarly assembled with strain-specific ONT reads. For assembly of the 45S rDNA cluster in *C. elegans* N2a, the right boundary was determined using the ONT reads containing both ETS and telomere sequences (TTAGGC). For *C. elegans* CB4856 45S rDNA cluster, the right boundary was determined using the ONT reads containing telomere sequences.

Reconstruction of the *C. briggsae* 5S rDNA cluster was started with two chromosome III contigs carrying a 5S rDNA sequence and genes next to rDNA sequences (*CBG06809* and *CBG10685*). The right boundary of 45S rDNA cluster was determined with the ONT reads carrying the rDNA sequence and those from its right boundary in CB4, which is located at the beginning of chromosome V. The 45S cluster left boundary was determined with the ONT reads carrying both 45S rDNA and telomere sequences.

### Draft genome assembly and quality assessment

To get a better reference genome for locating *C. briggsae* rDNA clusters, an AF16 draft genome was de novo assembled with ONT reads using Miniasm (v0.3). The resulting contigs were polished with Racon (v1.4.10) [49] two rounds using ONT reads and another three rounds using SLR reads [27]. Bacterial genomes were manually excluded from the polished contigs. Remaining 21 contigs were scaffolded into chromosomes using *C. briggsae* genome (CB4) as a reference and interspaced with 1000 “Ns”. The final draft genome was aligned against CB4 using LAST (v1021) [86]. The completeness of the resulting *C. briggsae* genome assembly, and the *C. elegans* N2 genome assembly (WBcel235) was assessed in parallel using BUSCO (v4.0.2) [50] with nematoda_odb10 database.

### Estimation of rDNA copy number

For estimation of copy number of *C. elegans* 5S rDNA units, the ONT reads mapped to the genomic interval of chrV: 17,110,000-17,430,000 (WBcel235) were extracted with SAMtools and were used for statistical analysis with SeqKit. The extracted reads were aligned against the 5S rRNA-coding sequence (referred to as 5S rRNA gene) with BLASTN with option “-word_size 7”. Sequences with alignment length > 17 bps were kept for the downstream analysis. The copy number of the 5S rDNA units was estimated for each library by dividing the summed read lengths aligned to 5S rRNA gene by the product between 5S RNA gene length (119) and genome-wide read coverage. For estimation of copy number of *C. elegans* 45S rDNA units, the reads mapped to the genomic interval of chrI: 15,057,500-15,072,434 (WBcel235) were extracted and aligned against the ITS1. Sequences with alignment length > 21 bps were kept for subsequent analysis. The copy number of the 45S rDNA units was estimated for each library by dividing the summed read lengths aligned to 45S rRNA gene sequence by the product between ITS1 length (464) and genome-wide read coverage.

For copy number estimation of *C. briggsae* 5S rDNA units, the reads mapped to the genomic interval of chrIII: 10,555,000-10,660,000 (*C. briggsae* CB4) were extracted. The extracted reads were aligned against the 5S rRNA gene sequence and two existing 5S rDNA units with BLASTN. Reads were retained for further analysis if the alignment size was bigger than 17, 170 and 170 bps for the 5S RNA gene, *cbr*-5S unit 1.1 and *cbr*-5S unit 2.1, respectively. The copy number of the 5S rDNA units was calculated in the same way as that in *C. elegans*. To extract all the reads mapped to the *C. briggsae* 45S rDNA cluster, a pseudo-chromosome was generated using chromosomal interval of chrI: 395,000-417,500 (CB4), which contains partial 26S rRNA gene and its flanking sequences consisting of five protein-coding genes and 100 copies of the *C. briggsae* 45S rDNA unit derived from SLR reads [27]. Reads mapped to the pseudo-chromosome were extracted and aligned against the *cbr*-ITS1 sequence with BLASTN. The copy number of the *C. briggsae* 45S rDNA unit was calculated in the same way as that in *C. elegans*.

### Validation of genomic localization and structure of assembled rDNA clusters

To validate the genomic localization of the assembled rDNA clusters in the *C. elegans* N2a and *C. briggsae* AF16, the Hi-C sequencing data from L1 stage animals [32, 87] were employed to confirm the linkage between the rDNA clusters and their host chromosomes. For the *C. elegans* reads, an rDNA pseudo-chromosome, which contains 50 copies of *cel*-5S unit 1.1 or 10 copies of *cel*-45S rDNA, was added into the reference genome for mapping of Hi-C reads. After trimming reads with Trimmomatic (v0.35) [88], the remaining reads were input to Juicer (v1.5) [89] with default parameters to find chromatin interactions between the rDNA pseudo-chromosome and host chromosomes. The density of interaction was normalized and visualized in R with circlize package (v0.4.7) [84, 90]. The linkage between the rDNA clusters and their host chromosomes in *C. briggsae* was performed in the same way as that in *C. elegans*. Specifically, the rDNA pseudo-chromosomes consisting of 50 copies of *cbr*-5S unit 1.1, or 50 copies of *cbr*-5S unit 2.1 and unit 2.2 with mixed arrangement, or 10 copies of *cbr*-45S rDNA, were individually added to the *C. briggsae* genome assembly CB4, respectively, for mapping of *C. briggsae* Hi-C reads [32].

To evaluate the structure of the newly assembled 5S rDNA clusters, the existing rDNA cluster sequences in the reference genome were replaced by the new assembly of rDNA sequences consisting of the minimum estimated copy number. The ONT reads were mapped against the modified reference genomes incorporated with the newly assembled rDNA cluster using Minimap2 with default parameters. The coverage within the new rDNA cluster was visualized in R with the ggplot2 package [83].

### Molecular biology, transgenesis, and imaging

All promoter fragments were amplified from N2a genomic DNAs with PCR primers listed in Table S2. The *miniMos* targeting vector pCFJ909 [45] was modified to include a genomic coding region of *his-24* that was fused to the GFP coding sequence at its 5′ end to facilitate nuclear localization, as previously described [91]. The fusion was cloned into the pCFJ909 vector, resulting in a reporter cassette consisting of the fusions: HIS-24::GFP or mCherry::HIS-24, which was followed by the sequence of *pie-1* 3′ UTR as described previously [92]. The vector was used for transgenesis with *miniMos* technique with the transgene insertion site being determined using inverse PCR [45]. All the micrographs were acquired with an inverted Leica SP5 confocal microscope equipped with two hybrid detectors at a constant ambient temperature of approximately 20 °C.

## Supplementary Information

**Additional file 1.**

**Additional file 2.**

**Additional file 3.**

**Additional file 4.**

**Additional file 5.**

**Additional file 6.**

**Additional file 7.**

**Additional file 8.**

**Additional file 9.**

**Additional file 10.**

**Additional file 11.**

## Data Availability

All base-called ONT reads from this study have been submitted to the NCBI BioProject database (https://www.ncbi.nlm.nih.gov/bioproject) under accession number PRJNA562392. The SRA and ENA accession number for each library is listed in Table S1. The sequences of rDNA units from this study were deposited in GenBank under accession number: MN519135 for *cel*-5S unit 1.1, MN519140 for *cel*-45S rDNA unit, MN519137 for *cbr*-5S unit 1.1, MN519138 for *cbr*-5S unit 2.1, and MN519141 for *cbr*-45S rDNA unit. Variant sequences for each rDNA unit and custom scripts for analyzing INDELs and SNPs of ONT reads with rDNA sequences were deposited in GitHub https://github.com/qiutaoding/QD_nanoKit_py3/tree/master/rDNA.

## Abbreviations

rDNAs: Ribosomal genes
ONT: Oxford Nanopore Technologies
CNVs: copy number variations
NGS: Next Generation Sequencing
NTS: non-transcribed sequences
Pol: RNA polymerase
SNPs: single-nucleotide polymorphisms
SL1: splicing leader
BAC: bacterial artificial chromosomes
SLR: synthetic long reads
SMRT sequencing: Single Molecule, Real-Time sequencing
ITS: internal transcribed spacers
ETS: external transcribed sequence
pSX1: positioning sequence on X
