# Supplementary material for: Genomic architecture of 5S rDNA cluster and its variations within and between species
Source: BMC Genomics. 2022 Mar 27;23:238. doi: 10.1186/s12864-022-08476-x (PMC8961926; doi:10.1186/s12864-022-08476-x)
Supplement: Supplementary file 1 — Additional file 1. [file 12864_2022_8476_MOESM1_ESM.pdf]

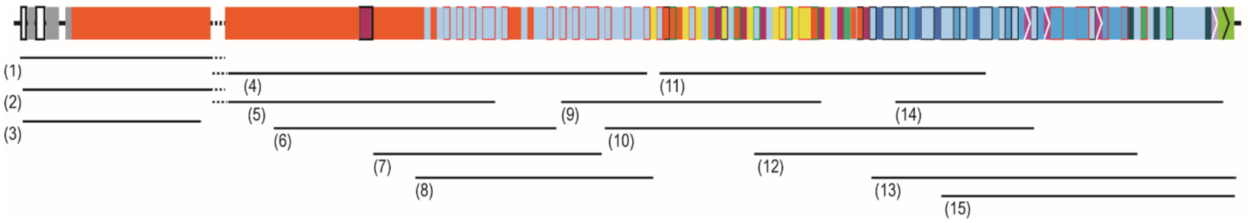

Fig. S1 Illustration of tiling path method for reconstructing 5S rDNA cluster in *C. elegans* N2a. The composition of 5S rDNA units are shown as color-coded bars as in Figure 1. The unsolved region (dash line) contains tandemly repeated *cel*-5S unit 1.1. Lines under the 5S rDNA cluster indicate the alignment of ONT reads. Read name and length are listed below. (1) 816e3c91-e978-404c-ad0c-0feac8208ddd, 63,658 bp. (2) 57556aff-f79a-4de4-81cc-d77ce3fec273, 48,846 bp. (3) 19aaf089-cf0c-4b24-9fae-2d14971bc320, 29,729 bp. (4) 28651bf0-8be1-4564-a740-ad17d0f260bd, 62,870 bp. (5) 77d84d3f-61ab-4fb1-9e4a-0fa291e6735d, 46,434 bp. (6) 594d2e66-ce5e-4b91-9f9e-7d6d24d9fbaa, 42,977 bp. (7) 7414a2d3-acdd-4c72-8509-e029ec1d7d13, 34,568 bp. (8) 367d1386-8328-4329-9ba6-c162993d8ede, 35, 021 bp. (9) 98b70228-6f3c-4e4b-ba71-b3631eea7acf, 38,624 bp. (10) 057d9497-6b73-4c82-9859-fd177a9930a2, 66,357 bp. (11) 9e96acd5-b022-4321-b922-bdbc7a33f7ab, 46,176 bp. (12) d42edfb5-7347-4c77-9d8c-23db3147a327, 50,719 bp. (13) 42938dc9-d10f-40cc-9558-cd6dade92882, 49, 434 bp. (14) c40285fc-c214-402d-b2c6-29b3d629c3f6, 52,513 bp. (15) bb18b8be-a27d-4119-aa91-b4b310f8cb28, 54, 031bp.

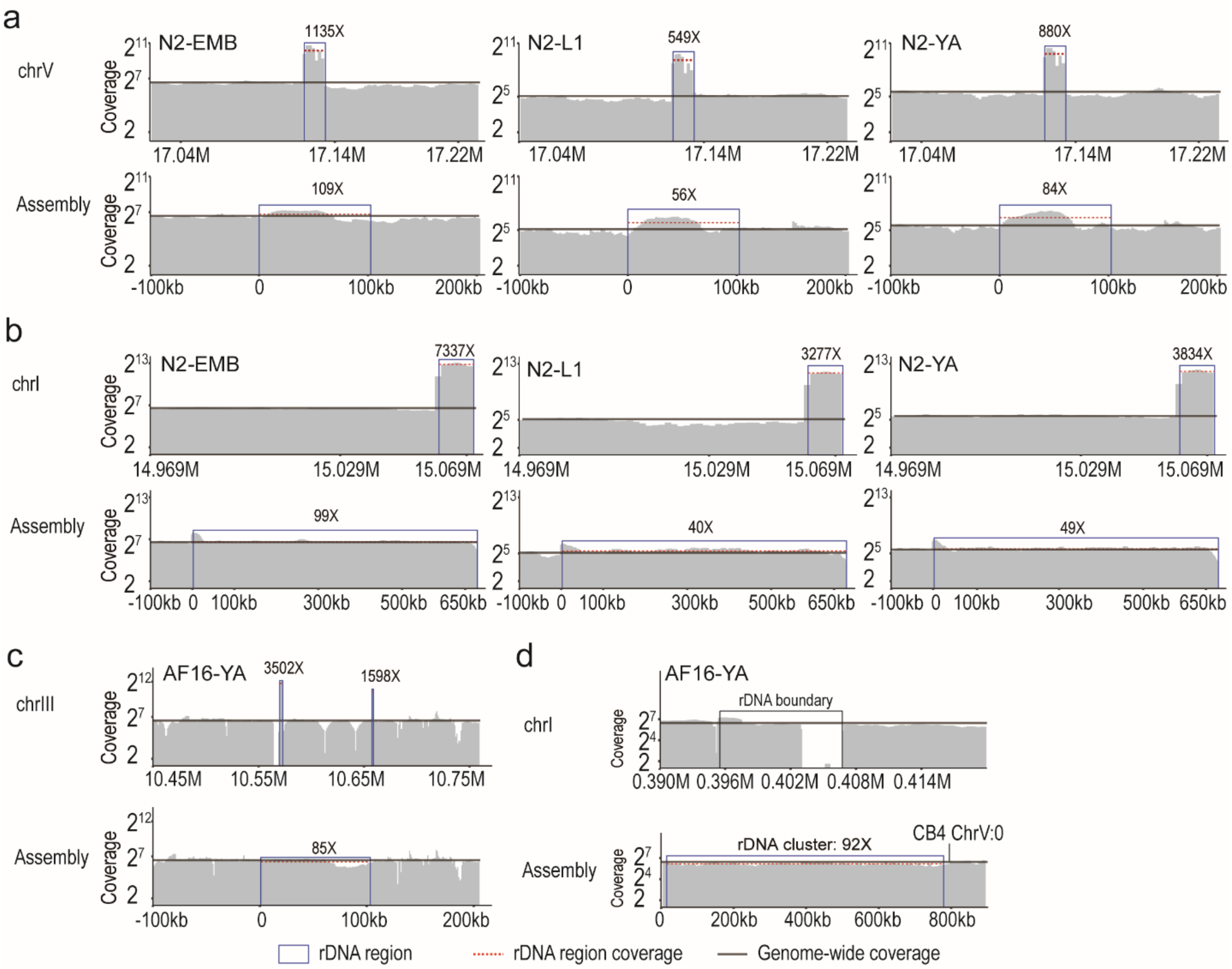

Fig. S2 Coverage changes in rDNA clusters. (a) The coverage changes between the *C. elegans* N2 reference chromosome V 5S rDNA cluster region and ONT reads assembled 5S rDNA cluster consensus. (b) The coverage changes between reference chromosome I 45S rDNA cluster region and ONT reads assembled 45S rDNA cluster consensus. (c) The coverage changes between the *C. briggsae* AF16 CB4 chromosome III 5S rDNA cluster region and the ONT reads assembled 5S rDNA cluster consensus. (d) The coverage of misassembled 45S rDNA cluster flanking sequences on CB4 chromosome I and the ONT reads assembled 45S rDNA cluster-containing region on chromosome V.

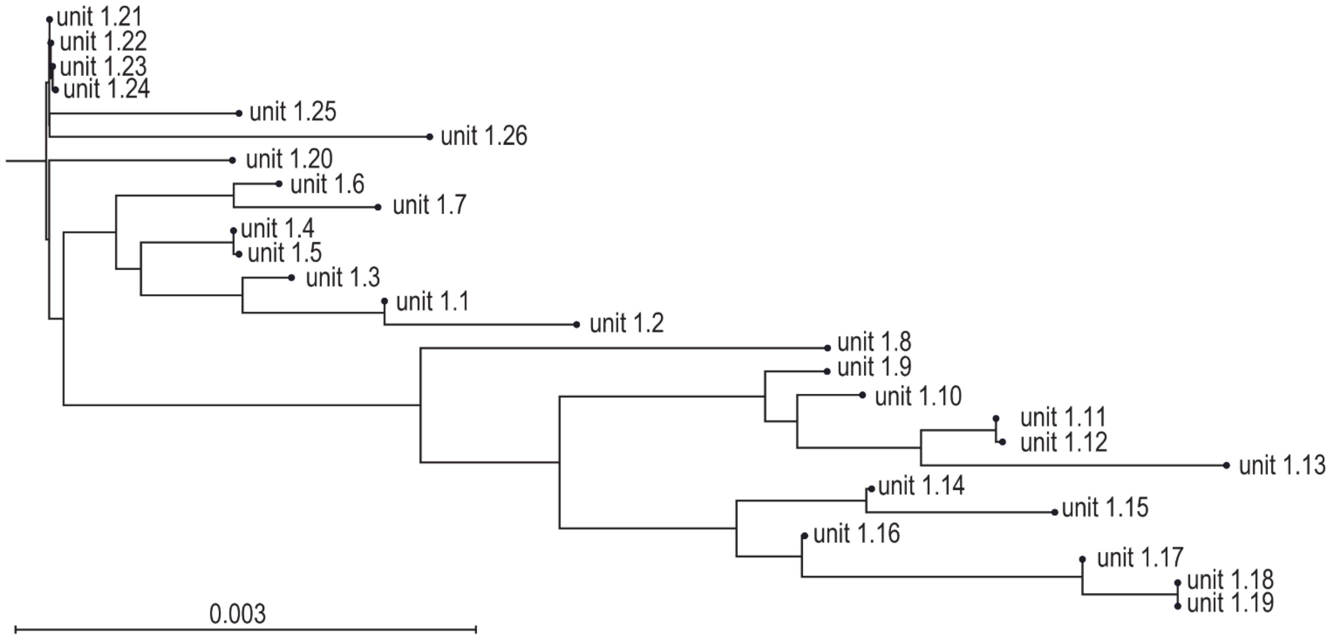

Fig. S3 Neighbor-joining phylogenetic tree of 5S rDNA units in N2a strain. Sequences of a total of 26 units carrying different variations were used for multiple sequence alignment. The units were named based on their sequence relatedness.

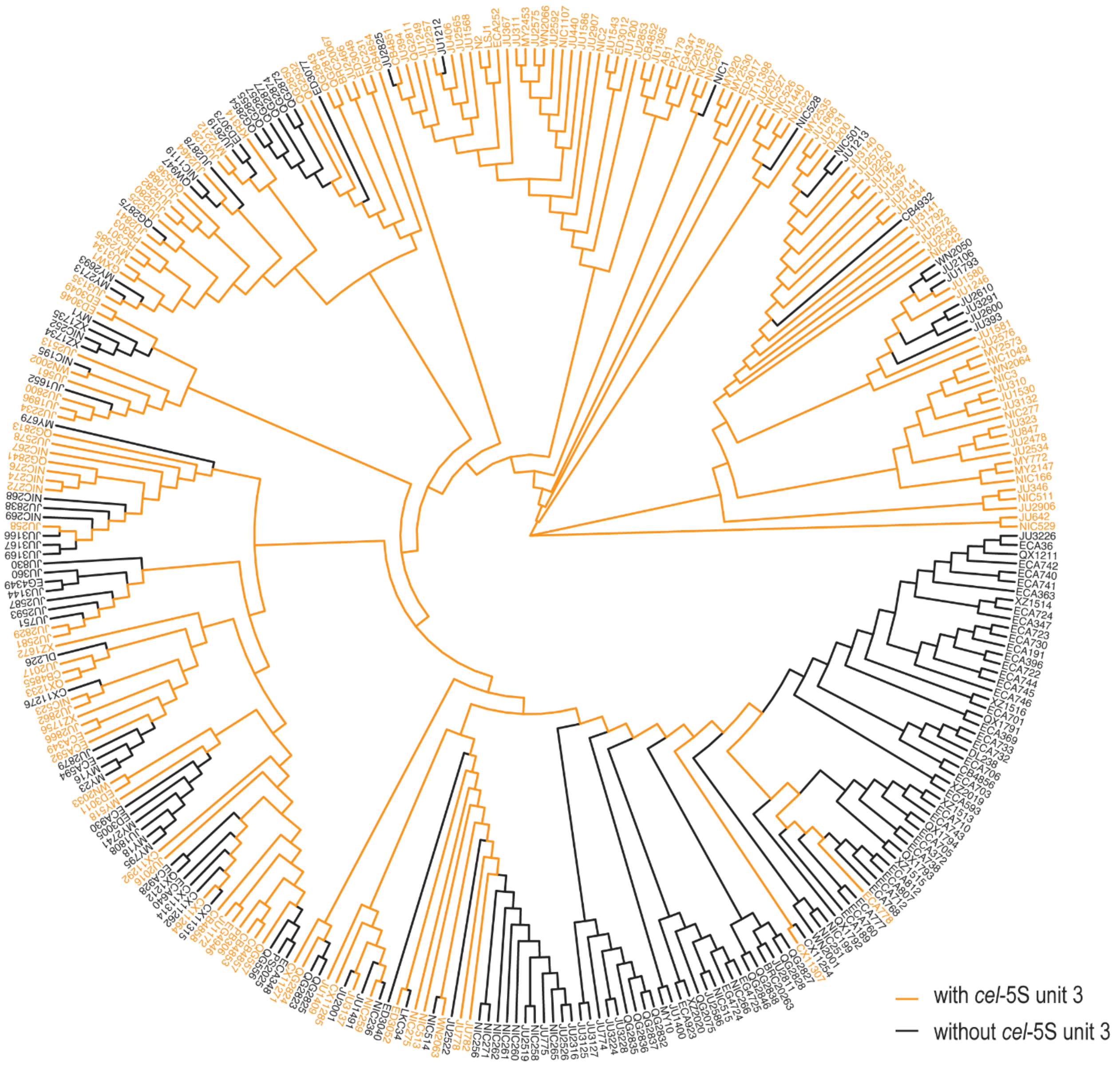

Fig. S4 Presence or absence of the 30-bp deletion in the 330 *C. elegans* wild-isolated strains shown in the phylogenetic tree generated previously (Cook *et al.* 2017)**.** Branches marked with black color indicate that the deletion is absent from NGS reads. See Table S6 for a detailed proportion of unique junctional reads carrying the deletion in each strain.

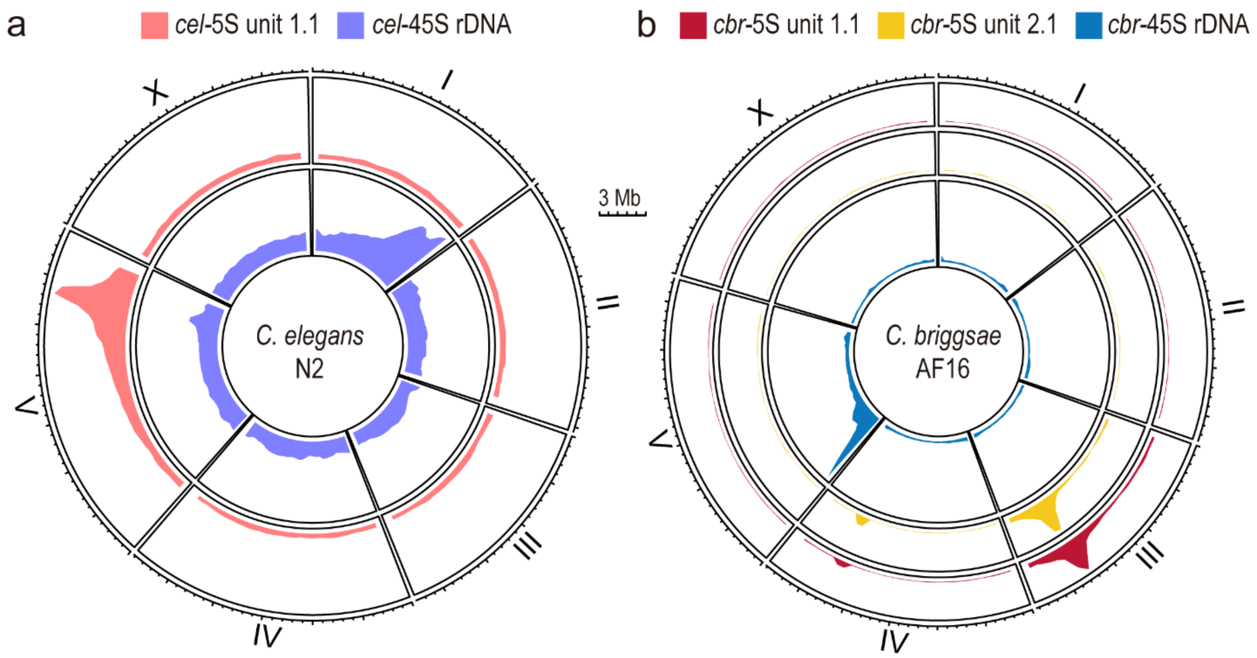

Fig. S5 Hi-C interactions between rDNA clusters and chromosomes. (a) The *C. elegans* N2 genomic linkage density between chromosomes and the 5S rDNA cluster (pink in the outer circle) and 45S rDNA cluster (purple in the inner cycle). (b) The *C. briggsae* AF16 genomic linkage density between chromosomes and pseudo-chromosomes of *cbr*-5S unit 1 (red in the outmost cycle), *cbr*-5S unit 2 (yellow in the middle cycle) and 45S rDNA (blue in the inner cycle).

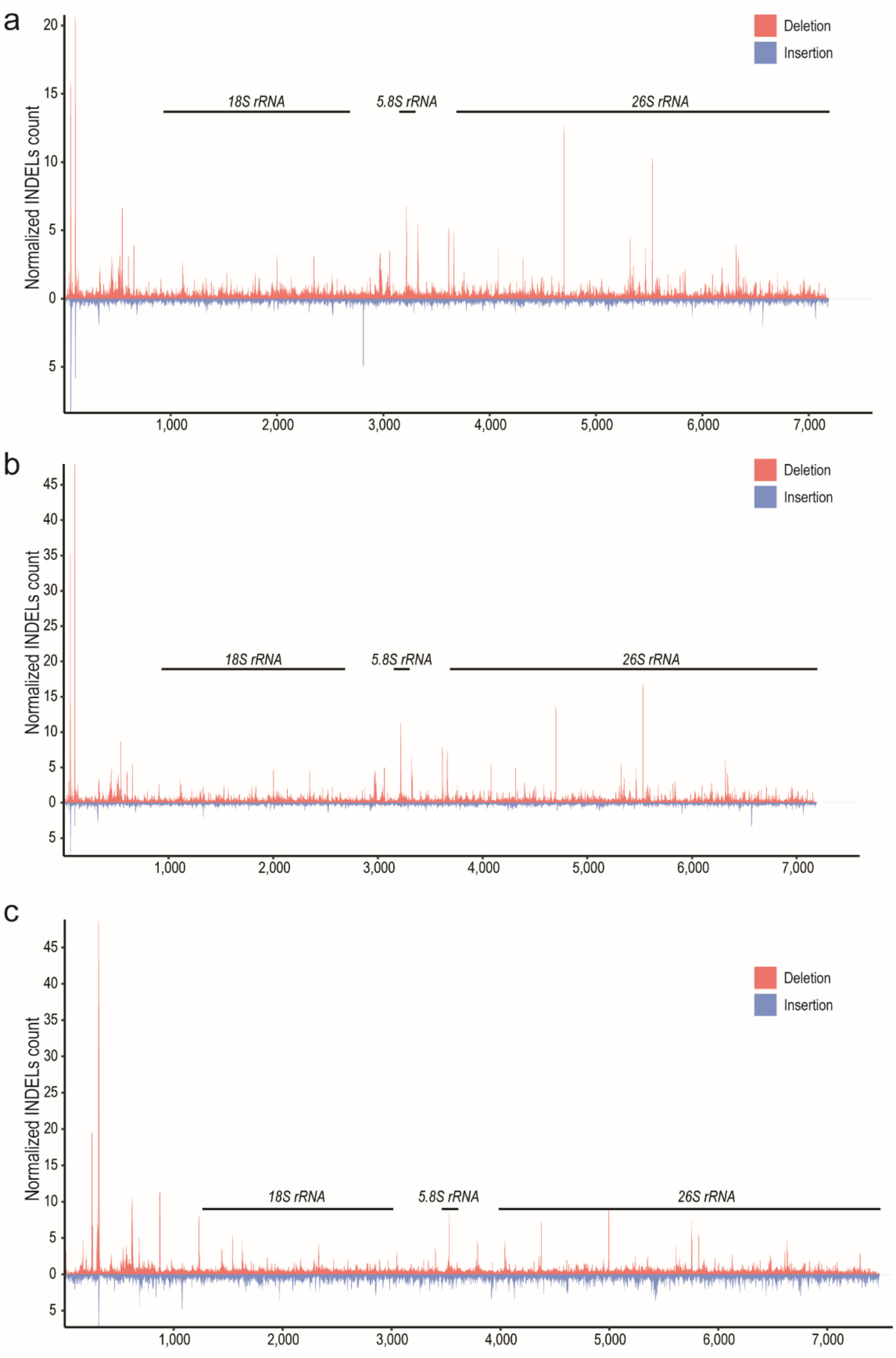

Fig. S6 Large INDELs in 45S rDNA cluster of *C. elegans* and *C. briggsae*. Histogram plots show the normalized INDELs count of ONT reads mapped to a single copy of 45S rDNA sequences in *C. elegans* N2 (a) and CB4856 (b), and *C. briggsae* AF16 (c) 45S rDNA clusters.

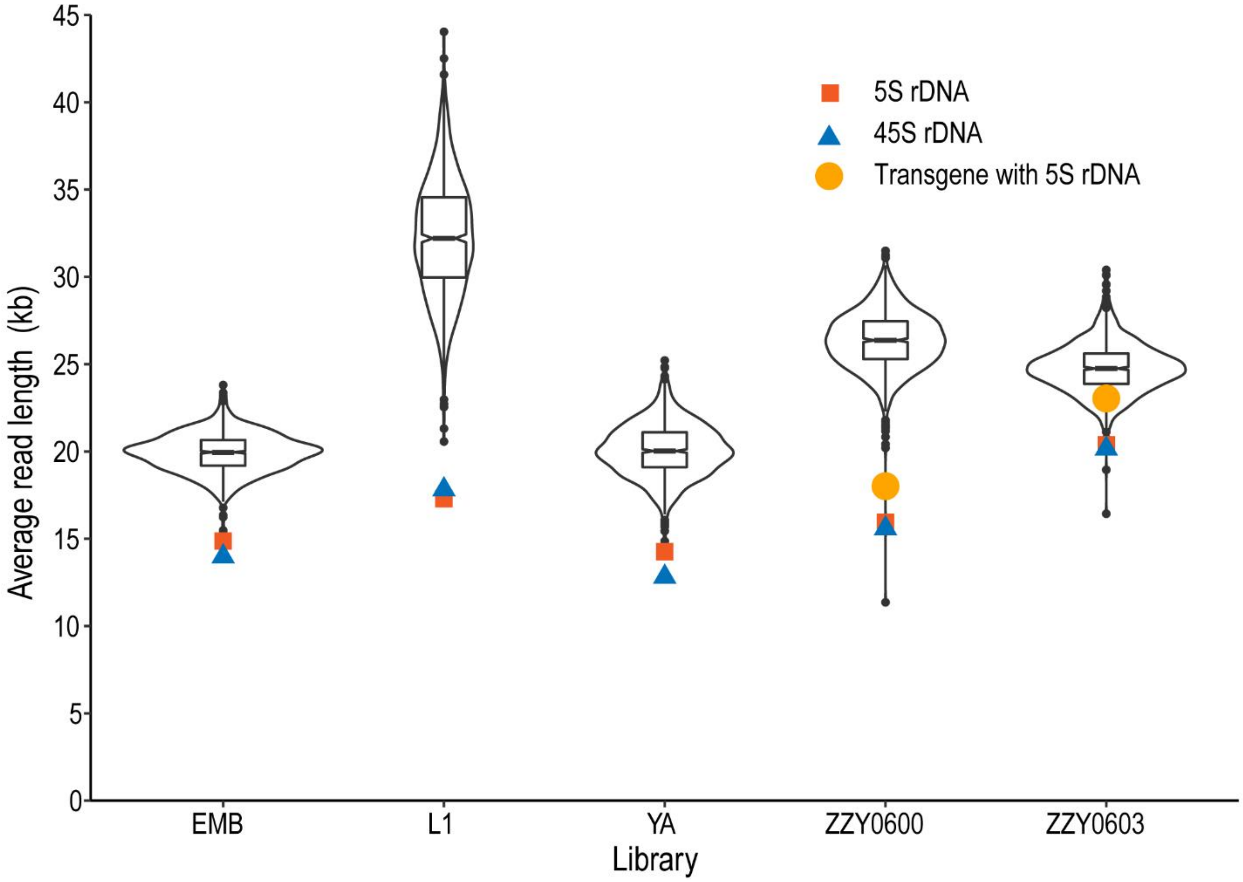

Fig. S7 Smaller average read length in the rDNA clusters than in other genomic loci. Distribution of average read length from 999 random positions over *C. elegans* genome and two rDNA clusters. Average lengths of ONT reads derived from 5S, 45S rDNA cluster, and the transgene-associated rDNA sequences are denoted in red square, blue triangle, and dark yellow, respectively. Box-plot shows the average read length ranging from Q1 quartile (25 percentiles) to Q3 quartile (75 percentiles) of ONT read length.

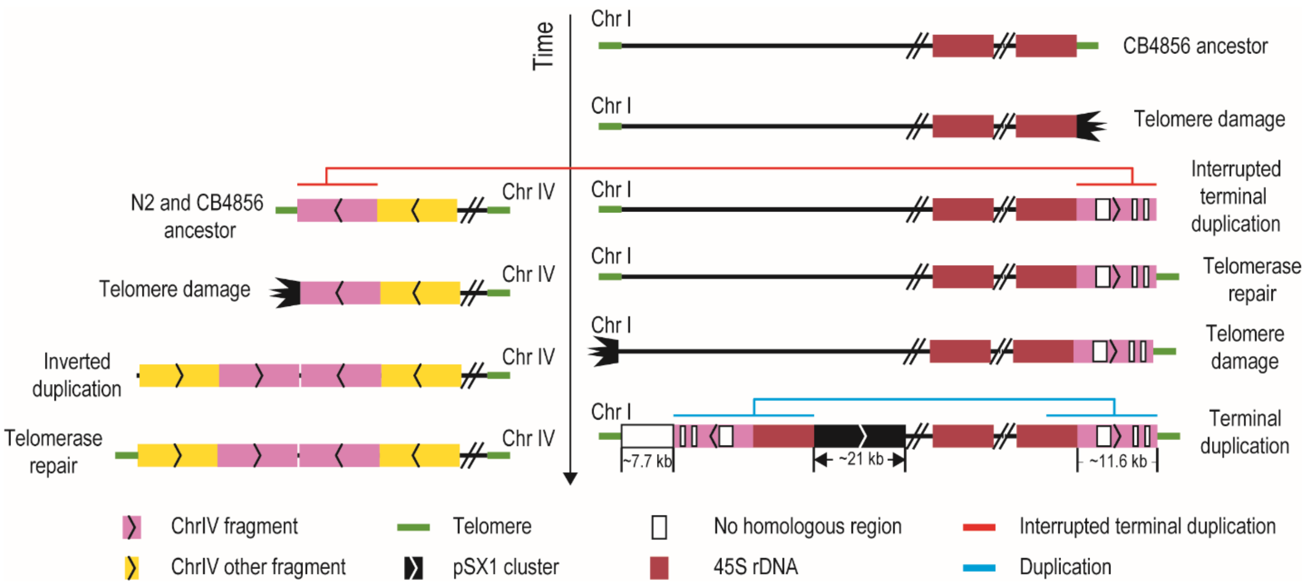

Fig. S8 A terminal duplication model of chrIL, chrIR, and chrIVL ends in *C. elegans* CB4856 ancestor. The CB4856 ancestor underwent telomere damage at chrIR end and sequential telomere-damage repair by interrupted terminal duplication from Chr IVL subtelomere and telomere lengthening. Afterward, the ancestor chrIVL underwent a telomere crisis and repaired by an inverted duplication with telomerase repairing. Then, the ancestor met telomere damage at chrIL end and sequential damage repair by the new chrIR terminal duplication including partial 45S rDNA. The chrIL pSX1 cluster containing 124 copies of full-length pSX1 (172 bp) and 5 partial-length copies is ~21 kb in length.

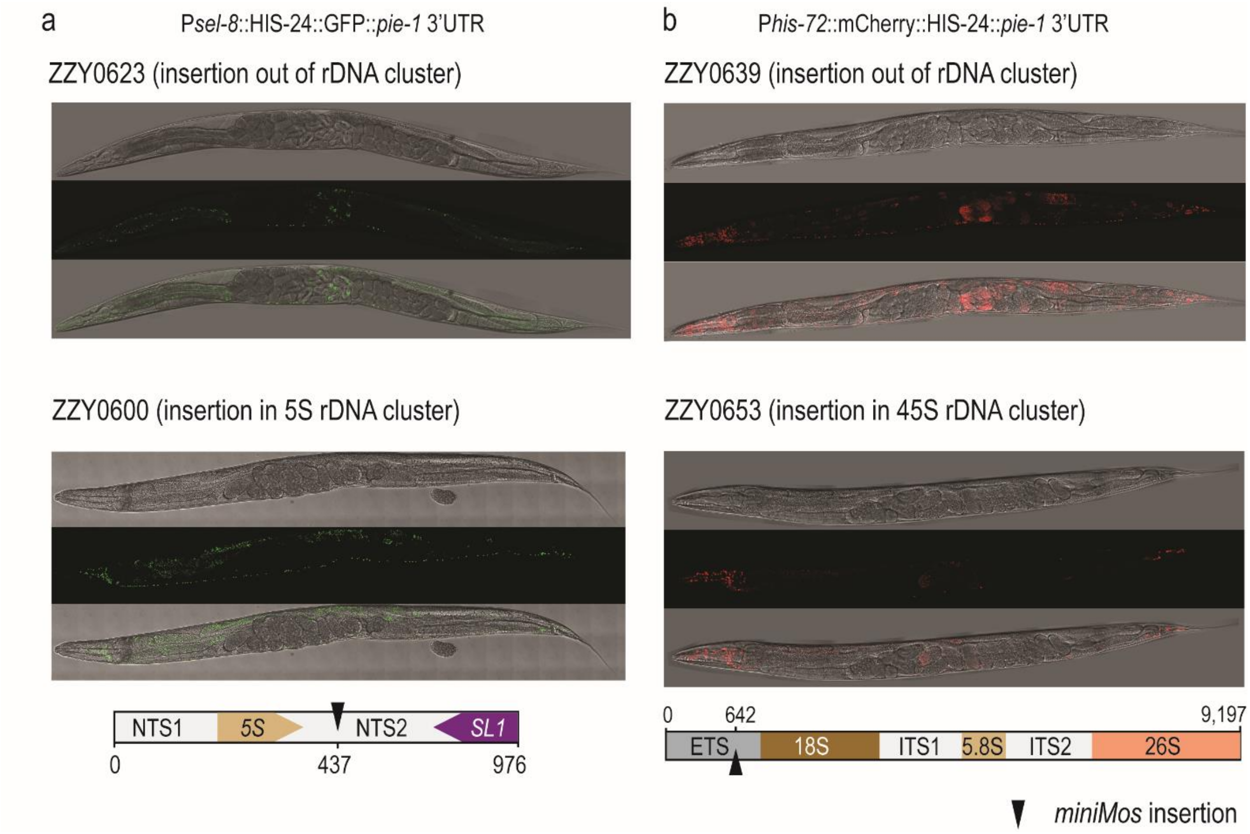

Fig. S9 Altered expression pattern of transgenes in rDNA clusters. (a) Comparison of the expression of transgene inserted inside and outside of 5S rDNA cluster: Outside: GFP expression is found in embryos with 350 or more cells, and head and tail cells and some of the neurons in adult animals in ZZY0623. Inside: GFP expression is found in the head and tail cells and neuron nucleuses in ZZY0600 adult worm. (b) Comparison of the expression of transgene inserted inside and outside of 45S rDNA cluster: Outside, mCherry expression is found in late-stage embryos, mitotic germline, and ubiquitously in ZZY0639 adult animals; Inside, mCherry expression is found in late-stage embryos and head and tail cells in ZZY0653 adult animals. The transgene cassette in ZZY0653 was inserted into ETS in the opposite direction to the unit.
